# An H3K27me3 demethylase-HSFA2 regulatory loop orchestrates transgenerational thermomemory in *Arabidopsis*

**DOI:** 10.1038/s41422-019-0145-8

**Published:** 2019-02-18

**Authors:** Junzhong Liu, Lili Feng, Xueting Gu, Xian Deng, Qi Qiu, Qun Li, Yingying Zhang, Muyang Wang, Yiwen Deng, Ertao Wang, Yuke He, Isabel Bäurle, Jianming Li, Xiaofeng Cao, Zuhua He

**Affiliations:** 1 National Key Laboratory of Plant Molecular Genetics, CAS Center for Excellence in Molecular Plant Sciences, Shanghai Institute of Plant Physiology & Ecology, Shanghai Institutes for Biological Sciences, Chinese Academy of Sciences, Shanghai 200032, China; 2 University of the Chinese Academy of Sciences, Beijing 100049, China; 3 School of Life Science and Technology, Shanghai Tech University, Shanghai 201210, China; 4 State Key Laboratory of Plant Genomics and National Center for Plant Gene Research (Beijing), Institute of Genetics and Developmental Biology, Chinese Academy of Sciences, Beijing 100101, China; 5 Shanghai Center for Plant Stress Biology, Chinese Academy of Sciences, Shanghai 201602, China; 6 Institute of Biochemistry and Biology, University of Potsdam, Potsdam, Germany; 7 Department of Molecular, Cellular, and Developmental Biology, University of Michigan, Ann Arbor, MI 48109, USA; 8 CAS Center for Excellence in Molecular Plant Sciences, Institute of Genetics and Developmental Biology, Chinese Academy of Sciences, Beijing 100101, China

**Keywords:** Plant signalling, Epigenetic memory, Innate immunity, Chromatin, Epigenetics

## Abstract

Global warming has profound effects on plant growth and fitness. Plants have evolved sophisticated epigenetic machinery to respond quickly to heat, and exhibit transgenerational memory of the heat-induced release of post-transcriptional gene silencing (PTGS). However, how thermomemory is transmitted to progeny and the physiological relevance are elusive. Here we show that heat-induced HEAT SHOCK TRANSCRIPTION FACTOR A2 (HSFA2) directly activates the H3K27me3 demethylase *RELATIVE OF EARLY FLOWERING 6* (*REF6*), which in turn derepresses *HSFA2*. REF6 and HSFA2 establish a heritable feedback loop, and activate an E3 ubiquitin ligase, SUPPRESSOR OF GENE SILENCING 3 (SGS3)-INTERACTING PROTEIN 1 (SGIP1). SGIP1-mediated SGS3 degradation leads to inhibited biosynthesis of *trans*-acting siRNA (tasiRNA). The REF6-HSFA2 loop and reduced tasiRNA converge to release *HEAT-INDUCED TAS1 TARGET 5* (*HTT5*), which drives early flowering but attenuates immunity. Thus, heat induces transmitted phenotypes via a coordinated epigenetic network involving histone demethylases, transcription factors, and tasiRNAs, ensuring reproductive success and transgenerational stress adaptation.

## Introduction

Increasing global temperatures have diverse and profound effects on plant growth, development and reproduction, and greatly threaten global crop yields.^1,2^ Plants have evolved sophisticated epigenetic machinery to respond quickly to heat.^3^ Heat exposure activates various transgenes and repetitive elements that are normally silenced via transcriptional gene silencing (TGS).^4–7^ Heat stress can also release post-transcriptional gene silencing (PTGS) by inhibiting siRNA biogenesis.^8–10^ Importantly, heat stress decreases the abundance of *trans*-acting siRNAs (tasiRNAs) derived from eight *Arabidopsis Trans*-acting siRNA (*TAS*) loci (*TAS1a-c*, *TAS2*, *TAS3a-c* and *TAS4*), triggering the upregulation of *HEAT-INDUCED TAS1 TARGET* (*HTT*) genes, which mediate thermotolerance by acting as cofactors of HEAT SHOCK PROTEIN 70–14 (Hsp70–14).^8^ It has been widely recognized that DNA methylation is not involved in the heat-induced release of gene silencing.^3^

Heat responses are usually transient and reset quickly to the normal conditions, although there are some somatic stress memory with the duration of several days.^11^ Two ATP-dependent chromatin remodeling factors, *Decrease in DNA methylation1* (*DDM1*) and *Morpheus’Molecule 1* (*MOM1*), have been shown to act in inhibiting the transgenerational transmission of heat-induced epigenetic states.^12^ Moreover, the transgenerational retrotransposition of *ONSEN* is prevented by the small interfering RNAs (siRNAs) pathway.^6^ However, certain plant responses to extreme or prolonged heat stress have been shown to exhibit transgenerational memory as they can be detectable in one or two subsequent stress-free generations.^7,10,13^ For instance, the immediate progeny of extreme heat shock (50 °C, 3 h/day for 5 days)-stressed *Arabidopsis* plants tend to bolt earlier.^13^ Heat stress (42 °C for 48 h)-mediated release of a reporter gene silencing can be transmitted to the non-stressed progeny, which was restricted to a small number of cells and limited to only two non-stressed progeny generations.^7^ Overall, the precise molecular mechanisms underlying the transgenerational memory of heat stress in plants remain poorly understood and disputed.

We previously reported that prolonged high temperature (30 °C for 13 days) led to deterministic suppression and transgenerational inhibition of PTGS and tasiRNA biogenesis in *Arabidopsis*.^10^ Our data suggested that heat stress reduces the steady-state level but not the transcription level of SUPPRESSOR OF GENE SILENCING 3 (SGS3), a plant-specific RNA binding protein critical for siRNA biogenesis.^14^ However, how the memory of heat stress is established and transmitted to progeny and whether other phenotypes are affected remained largely unknown.

Here, we discover that HEAT SHOCK TRANSCRIPTION FACTOR A2 (HSFA2) and H3K27me3 demethylase RELATIVE OF EARLY FLOWERING 6 (REF6) form a positive feedback loop to transmit long-term epigenetic memory of heat by maintaining the transgenerational “ON state” of *HSFA2*, which activates an F-Box E3 ubiquitin ligase gene, *SGS3-INTERACTING PROTEIN 1* (*SGIP1*), to execute the transgenerational degradation of SGS3. More importantly, the thermomemory machinery promotes plant reproduction and fitness by accelerating flowering and attenuating disease resistance through the tasiRNA target *HTT5*.

## Results

### Transgenerational memory of early flowering and attenuated immunity via suppression of tasiRNAs

**Fig. 1:**
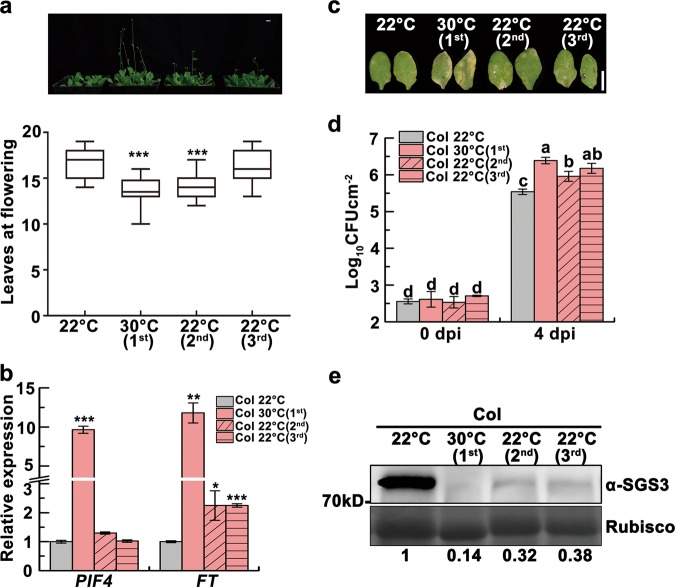
Heat-induced transgenerational degradation of SGS3 accelerates flowering but attenuates immunity. **a** Four-week-old 22 °C-grown Col, heat-stressed (first) and unstressed second and third generation plants and box plots of flowering times of these four lines. Flowering time was assessed by counting total leaf numbers in bolting plants (*n* ≥ 15 for each line). Bars represent means ± SD. **b** *PIF4* and *FT* transcript levels as normalized to the *ACTIN2* signals. The average values (±SD, *n* = 3) were shown. Samples were collected from 17-day-old seedlings. Asterisks indicate significant difference (Student’s *t-*test; *P < 0.05, **P < 0.01, ***P < 0.001) (**a**, **b**). **c** Disease symptoms at 4 dpi with *Pst* DC3000 (*avrRpt2*). Scale bar, 1 cm (**a**, **c**). **d** Bacterial growth in plants in **c** was measured at 0 and 4 dpi. Bars represent means ± SD (*n* = 3). Two-way ANOVA with Tukey’s HSD post hoc test (significance set at *P* < 0.05) was performed. Different lowercase letters indicate significant differences. **e** SGS3 levels were immunodetected, with relative levels shown under lanes. Samples were collected from 24-day-old 22 °C-grown Col, heat-stressed (first) and early flowering, unstressed second and third generation plants. Rubisco served as a loading control

Extreme heat (50 °C)-stressed plants show accelerated flowering, which is also observed in the immediate unstressed progeny, however, the underlying mechanism remains unknown.^13^ To investigate whether the prolonged high temperature (30 °C) has similar effect in the progeny, we generated prolonged heat-stressed seedlings (Supplementary information, Fig. S1a) and analyzed the flowering time in their progeny. Interestingly, we found that parental (first generation) Col plants grown at prolonged high temperature bolted earlier than those grown at 22 °C, and that most of their unstressed second generation progeny also blotted earlier (Fig. 1a). Although there was no statistical significance, part of third generation progeny still tended to bolt earlier (Fig. 1a), similar to the heat-induced PTGS release which was maintained for at least three generations with rapidly declining strength.^10^ High temperatures accelerate flowering via induction of *PHYTOCHROME INTERACTING FACTOR4* (*PIF4*), which activates florigen *FLOWERING LOCUS T* (*FT*).^15^ Indeed, *FT* was upregulated in heat-stressed wild-type Col and unstressed progeny both before and after blotting (Fig. 1b; Supplementary information, Fig. S1b). In contrast, although high temperatures induced *PIF4* expression, *PIF4* upregulation was not detected in unstressed progeny, indicating that factors other than *PIF4* are involved in the transgenerational thermomemory (Fig. 1b; Supplementary information, Fig. S1b, c).

High temperatures are known to diminish the resistance to bacterial pathogens through inhibiting the salicylic acid (SA) defense pathway or upregulating *PIF4*.^16,17^ Interestingly, we observed transgenerational memory of heat-induced enhanced susceptibility to avirulent *Pseudomonas syringae* (*P. syringae*) *Pst* DC3000 (*avrRpt2*) (Fig. 1c, d). Thus, heat stress induces a memory of attenuated immunity, along with early flowering and PTGS release. Since *PIF4* is not transgenerationally upregulated (Fig. 1b; Supplementary information, Fig. S1b, c), we then investigated the SA pathway. Inoculated with *Pst* DC3000 (*avrRpt2*), heat-stressed plants showed much lower inductions of the SA biosynthesis gene *ISOCHORISMATE SYNTHASE 1* (*ICS1*), the SA signaling marker *PATHOGENESIS-RELATED GENE 1* (*PR1*), and SA, but their levels were partially restored in unstressed progeny (Supplementary information, Fig. S2a–c).

Since heat stress reduces the levels of SGS3 (Fig. 1e) and tasiRNAs,^10^ we hypothesized that reduced SGS3 and tasiRNA levels might result in the transgenerational memory of early flowering and attenuated immunity, similar to PTGS release.^10^ We observed reduced levels of endogenous SGS3, siR255 and siR1511 (two widely studied tasiRNAs that derive from *TAS1* and *TAS2*, respectively)^18^ in heat-stressed first generation Col plants, and in early flowering unstressed second and third generation plants (Fig. 1e; Supplementary information, Fig. S3a). We also found that the loss-of-function *sgs3–12* mutant^19^ and *MIM480(+)* plants, which have reduced levels of *TAS1*-siRNAs,^8^ also bolted earlier when grown at 22 °C and were not affected by heat, associated with upregulated *FT* (Supplementary information, Fig. S3b, c). These results suggest that the heat-induced decrease in tasiRNAs is involved in the thermomemory of early flowering.

We next examined whether the reduced SGS3 and tasiRNA levels also contribute to the transgenerational memory of attenuated immunity. Indeed, *sgs3–12* mutant and *MIM480(+)* plants were more susceptible to *Pst* DC3000 (*avrRpt2*) relative to wild-type plants (Supplementary information, Fig. S3d), with only slightly reduced induction of *ICS1* and SA levels upon pathogen infection (Supplementary information, Fig. S3e, f). These results suggest that depletions of SGS3 and tasiRNAs compromise immunity, which may be only partially dependent on the SA pathway. Thus, the heat-induced memory of attenuated immunity is likely caused by defects in multiple defense pathways. Based on these data, we propose that thermomemory affects the fitness of stressed plants and their unstressed progeny by accelerating reproductive development associated with attenuated immunity through certain tasiRNA target(s), consistent with the trade-off between growth and defense.^20,21^

### SGIP1 targets SGS3 for degradation

Our findings so far suggest that heat stress triggers transgenerational inhibition of SGS3 (Fig. 1e). In a cell-free degradation assay, we observed faster degradation of SGS3 isolated from heat-stressed seedlings, and delayed SGS3 degradation with treatment of the proteasome inhibitor MG132 (Supplementary information, Fig. S4a). The heat-enhanced degradation of SGS3 was further confirmed in Col seedlings treated with cycloheximide (CHX), which can block new protein synthesis, and MG132 treatment inhibited SGS3 degradation both at 22 °C and 30 °C (Supplementary information, Fig. S4b). These results suggest that a heat-upregulated E3 ligase targets SGS3 for degradation. Therefore, we analyzed published data^22,23^ and pursued 46 putative heat-responsive E3 ligases, which could be induced upon heat treatment (Supplementary information, Table S1).

**Fig. 2:**
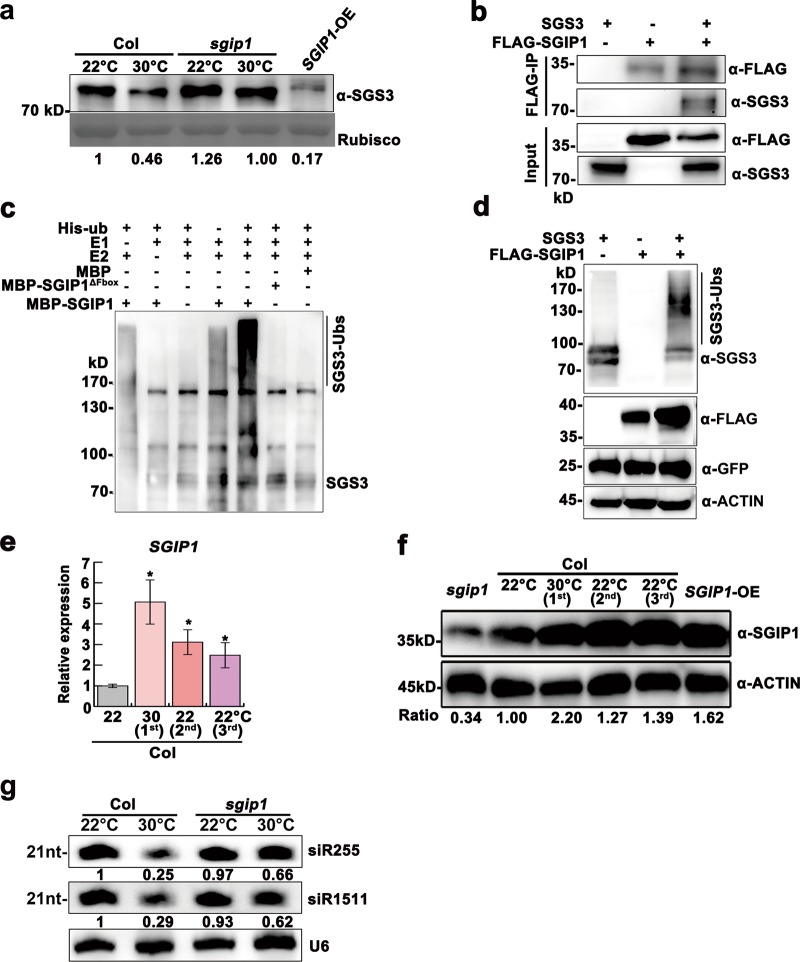
*SGIP1* mediates thermomemory degradation of SGS3 and tasiRNA suppression. **a** Immunodetection of SGS3 abundance in 24-day-old plants. Coomassie blue staining of Rubisco served as a loading control. **b** Co-IP assays of SGIP1 with SGS3 in *N. benthamiana* leaves. Proteins were immunoprecipitated with an anti-FLAG antibody, and detected by western blot using the anti-SGS3 and anti-FLAG antibodies. **c** In vitro ubiquitination of SGS3 by SGIP1. Polyubiquitination of SGS3 by SGIP1 was detected by immunoblotting using the anti-SGS3 antibody. The mutated SGIP1 protein (SGIP1^△Fbox^) served as a negative control. **d** SGIP1 promotes SGS3 polyubiquitination and degradation in vivo. Co-expression of *SGIP1* and *SGS3* was performed in *N. benthamiana* leaves. Protein accumulation of SGS3 and FLAG-SGIP1 was immunodetected with the anti-SGS3 and anti-FLAG antibody, respectively. GFP was co-expressed as an internal control. **e** Expression levels of *SGIP1* as normalized to the *ACTIN2* signals. Error bars indicate the SD (*n* = 3). Asterisks indicate significant difference between lines and 22 °C-grown Col (Student’s *t-*test; *P < 0.05, **P < 0.01). **f** Immunodetection of SGIP1 levels with an anti-SGIP1 antibody. ACTIN served as a loading control (**d**, **f**). **g** Knockdown of *SGIP1* maintained the production of siR255 and siR1511 at 30 °C. U6 was used as a loading control. The intensity of the blots was quantified (**a**, **f**, **g**)

We screened the homozygous mutants of 46 putative heat-responsive E3 ligases at 22 °C and 30 °C and identified that a knockdown mutant of *AT3G47020* showed impaired heat-induced decrease in SGS3 abundance (Fig. 2a; Supplementary information, Fig. S4c), and delayed SGS3 degradation compared to Col in vivo and in vitro (Supplementary information, Fig. S4d, e), leading us to investigate the possibility that this E3 ligase interacts with SGS3 to trigger its degradation. We found that AT3G47020 and SGS3 co-localized in the cytoplasmic granules (Supplementary information, Fig. S5a), and interacted in the yeast two-hybrid assay and the split luciferase complementation assay (Supplementary information, Fig. S5b, c). Importantly, SGS3 co-immunoprecipitated with FLAG-AT3G47020 in planta (Fig. 2b). These results suggest that AT3G47020 physically interacts with, and may directly regulates, SGS3. Thus, we refer to this putative E3 as SGS3-INTERACTING PROTEIN 1 (SGIP1) and the knockdown mutant of *AT3G47020* as *sgip1*.

To test whether SGIP1 has E3 ligase activity, we expressed and purified an MBP-SGIP1 fusion protein from *E. coli*. MBP-SGIP1 can polyubiquitinate SGS3 immunopurified from *N. benthamiana* (Fig. 2c). Further, we detected polyubiquitinated SGS3 in *N. benthamiana* leaves co-expressing SGS3 with FLAG-SGIP1 (Fig. 2d). These results reveal that SGIP1 targets SGS3 for ubiquitination.

*SGIP1* expression was upregulated in heat-stressed plants and unstressed progeny (Fig. 2e, f). To determine if *SGIP1* upregulation is sufficient to induce SGS3 degradation, we engineered Col plants overexpressing SGIP1 (*SGIP1*-OE) (Fig. 2f; Supplementary information, Fig. S4c), which displayed elongated, downwardly curled leaves, bolted earlier and showed enhanced disease susceptibility, similar to *sgs3–12* mutant plants (Supplementary information, Figs. S3b, d and S6a-c). Importantly, *SGIP1-*OE plants had substantially reduced levels of SGS3, siR255 and siR1511 (Fig. 2a; Supplementary information, Fig. S6d). Further, *AT1G62590*, *AUXIN RESPONSE FACTOR4* (*ARF4*) and *MYB DOMAIN PROTEIN 75* (*MYB75*), which are targeted by *TAS2*, *TAS3* and *TAS4*-derived tasiRNAs, respectively,^24,25^ were upregulated in *SGIP1*-OE plants, as in *sgs3–12* plants (Supplementary information, Fig. S6e). In contrast, similar to *p35S::SGS3-GFP* plants reported previously,^10^ heat-stressed *sgip1* plants neither bolted earlier (Supplementary information, Fig. S6a, b), nor showed reduced siR255 and siR1511 levels (Fig. 2g), but showed diminished upregulation of the tasiRNA targets (Supplementary information, Fig. S6e). Thus, SGIP1 is a heat-induced negative regulator of SGS3 and, in turn tasiRNAs. Collectively, the results reveal that SGIP1 targets SGS3 for ubiquitin-dependent degradation, and that the heat-induced activation of *SGIP1* is inherited in progeny. The *SGIP1-*OE plants blotted earlier, while the flowering time of *sgip1* mutant plants showed no thermomemory, suggesting that SGIP1 is important for transgenerational thermomemory.

### H3K27me3 demethylation confers transgenerational upregulation of *HSFA2* and its target *SGIP1*

**Fig. 3:**
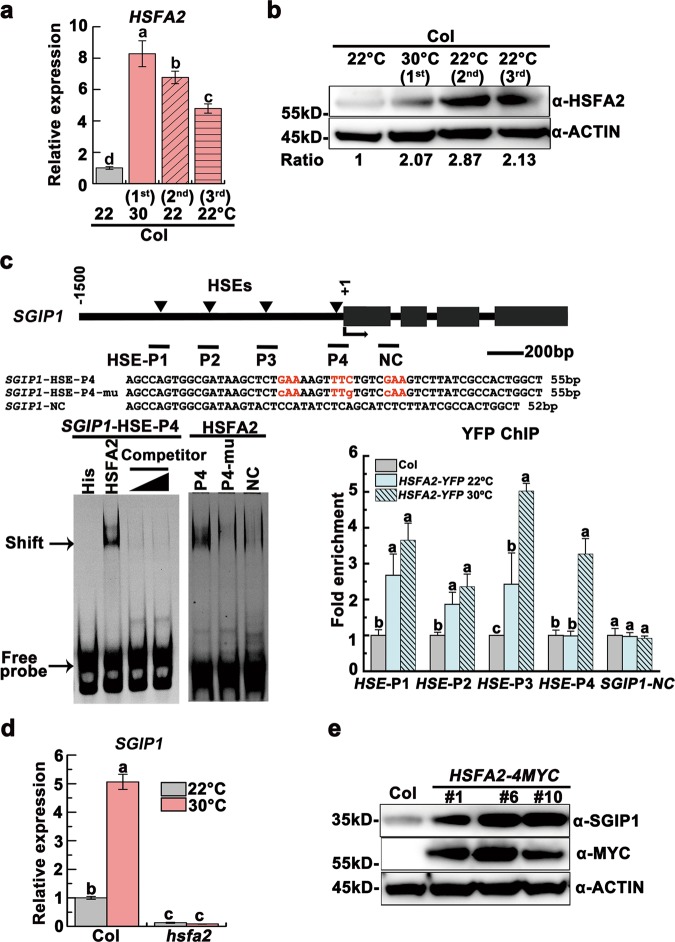
HSFA2 activates *SGIP1* transgenerationally through binding to its promoter. **a**, **b** *HSFA2* remained upregulated in the progeny of heat-stressed plants at both transcript (**a**) and protein (**b**) levels. **c** HSFA2 binds to the *SGIP1* promoter HSEs in vitro and in vivo. Left: EMSA shows the direct binding of His-HSFA2 to the HSE-P4 of *SGIP1*. The arrow indicates the shifted bands. Excess unlabeled probe (500 ng and 1000 ng) outcompeted the labeled probe (right lanes). Sequence of the probe is shown and the motif is highlighted in red. A mutant HSE and a downstream sequence *SGIP1-NC* (negative control) were used as negative controls. Right: ChIP-qPCR validation of HSFA2 occupancy at the HSE regions of the *SGIP1* promoter. Data are shown as relative fold enrichments over the background (Col). The *SGIP1-NC* locus was used as the negative-control locus. The locations of 4 HSEs are indicated by triangles above the gene model. The regions validated by ChIP-qPCR were marked by a bar below the gene model. **d** *SGIP1* transcript levels in Col and *hsfa2* plants grown at 22 °C and 30 °C. Gene expression was shown as mean ± SD (*n* = 3). *ACTIN2* was analyzed as an internal control (**a**, **d**). Lowercase letters indicate significant differences, as determined by the post hoc Tukey’s HSD test (**a**, **c**, **d**). **e** The abundance of SGIP1 protein increased in three transgenic lines overexpressing *HSFA2-MYC*, as immunodetected with anti-SGIP1 and anti-MYC antibodies. ACTIN served as a loading control (**b**, **e**) and the signals were quantified (**b**)

We noticed that the *SGIP1* promoter contains four HSF-targeting heat shock elements (HSE).^26^ We hypothesized that HSFs regulate *SGIP1*. Indeed, *HSFA2*, *HSFA3* and *HSFA7a* were obviously upregulated at 30 °C (Supplementary information, Fig. S7a), and *HSFA2*, a major heat stress transcription factor,^3,26^ remained upregulated in the unstressed progeny (Fig. 3a, b; Supplementary information, Fig. S7b). Further, we detected direct binding of HSFA2 to the *SGIP1* promoter HSEs in vitro (Fig. 3c; Supplementary information, Fig. S7c). In addition, ChIP-qPCR of *hsfa2* knockout plants expressing a *pHSFA2::HSFA2-YFP* fusion revealed that HSFA2-YFP bound to *SGIP1* P1-P3 at 22 °C, and this binding increased and extended to P4 under heat stress (Fig. 3c). The expression of *SGIP1* was dramatically decreased in *hsfa2* plants (Fig. 3d), whereas transgenic plants overexpressing HSFA2-MYC displayed early flowering as well as elevated SGIP1 and a concomitant decrease in SGS3 (Fig. 3e; Supplementary information, Fig. S7d, e). These data suggest that upregulation of *HSFA2* in heat-stressed plants and unstressed progeny leads to direct activation of *SGIP1*.

**Fig. 4:**
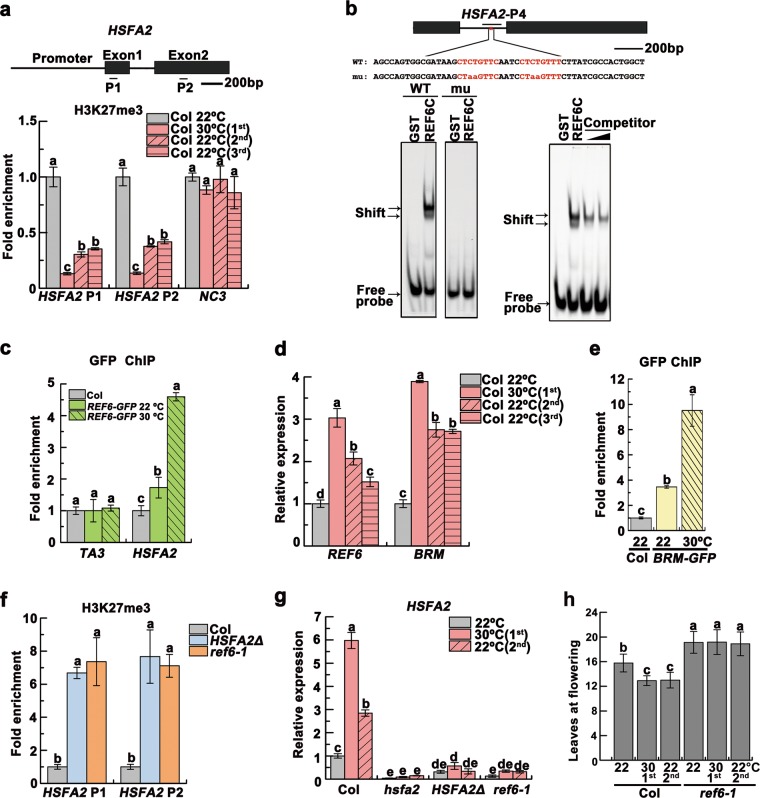
H3K27me3 demethylation controls transgenerational upregulation of *HSFA2*. **a** H3K27me3 levels detected by ChIP-qPCR at the *HSFA2* loci. The gene model was shown and the analyzed regions (P1 and P2) were indicated. One intergenic region (NC3) without H3K27me3 mark^28^ was used as negative background. **b** EMSA showed *HSFA2* probe binding to GST-REF6C containing the C2H2-ZnF cluster. The mutant probe abolished the binding of GST-REF6C. Excess unlabeled probe outcompeted the labeled probe (right panel). The motif is highlighted in red in the probe sequence, with mutated bases shown in lowercase. The region validated by ChIP-qPCR in **c** was marked by a bar. **c** ChIP-qPCR validation of REF6 binding at *HSFA2* using the *REF6-GFP* plants. Col was used as the negative control. The *TA3* locus was used as the negative-control locus. **d** Transcript levels of *REF6* and *BRM*, shown as mean ± SD (*n* = 3). **e** ChIP-qPCR validation of BRM binding at *HSFA2* using the *BRM-GFP* plants. Col was used as the negative control. **f** H3K27me3 levels of *HSFA2* in the *HSFA2Δ* and *ref6–1* plants. The data were normalized to the corresponding input fraction (**a**, **c**, **e**, **f**). **g** *HSFA2* transcript levels. Data were shown as means ± SD from three replicates (**a**, **c–g**). **h** Flowering times of indicated lines were assessed (*n* ≥ 15 for each line). Lowercase letters indicate statistical significance based on one-way (**a**, **c–f**) or two-way (**g**, **h**) ANOVA with Tukey’s HSD post hoc analysis (*P* < 0.05)

We previously found that changes in DNA methylation did not correlate with the heat-induced transgenerational release of PTGS.^10^ To investigate whether other epigenetic mechanisms contribute to the regulation of *HSFA2*, we evaluated various histone modifications, and found that unstressed progeny inherited a heat-induced depletion of H3K27me3 within the coding regions of *HSFA2* (Fig. 4a), which may contribute to the upregulation of *HSFA2* in heat-stressed plants and unstressed progeny.

REF6 recognizes a CTCTGYTY (Y = T or C) motif to erase H3K27me3.^27,28^ We identified two of these motifs within *HSFA2* intron 1. We found that REF6 bind to the *HSFA2* region in vitro (Fig. 4b), and validated the binding of REF6 in vivo by ChIP-qPCR (Fig. 4c). *REF6* was upregulated by heat and its activation was maintained transgenerationally (Fig. 4d). REF6 recruits the chromatin remodeler BRAHMA (BRM) to activate gene expression.^27^ Consistent with this, *BRM* showed heat-induced transgenerational upregulation and the binding of BRM to the *HSFA2* region was enhanced by heat (Fig. 4d, e). To evaluate the role of REF6/BRM binding in the transgenerational upregulation of *HSFA2*, we used the clustered regularly interspaced short palindromic repeat (CRISPR)/CRISPR-associated 9 (Cas9) technology to delete a 104-bp fragment containing the two REF6 binding motifs from *HSFA2* intron 1 (Supplementary information, Fig. S8a). Similar to *ref6–1* mutants, the *HSFA2Δ* plants displayed elevated H3K27me3 levels and reduced expression of *HSFA2* (Fig. 4f, g), suggesting that these motifs directly recruit REF6/BRM to regulate H3K27me3 levels and *HSFA2* expression. The flowering time of *ref6–1* mutant plants showed no thermo-responsiveness or thermomemory (Fig. 4h), suggesting that the thermomemory of early flowering is dependent on the H3K27me3 epigenetic regulation. Intriguingly, *hsfa2* and *HSFA2Δ* plants also trended early flowering, but they likely lost the thermomemory on early flowering (Supplementary information, Fig. S8b). Together, these data indicate that heat exposure reduces H3K27me3 at *HSFA2* via REF6/BRM, which is inherited in unstressed progeny.

### A heritable REF6-HSFA2 feedback loop enables cooperative gene activation

**Fig. 5:**
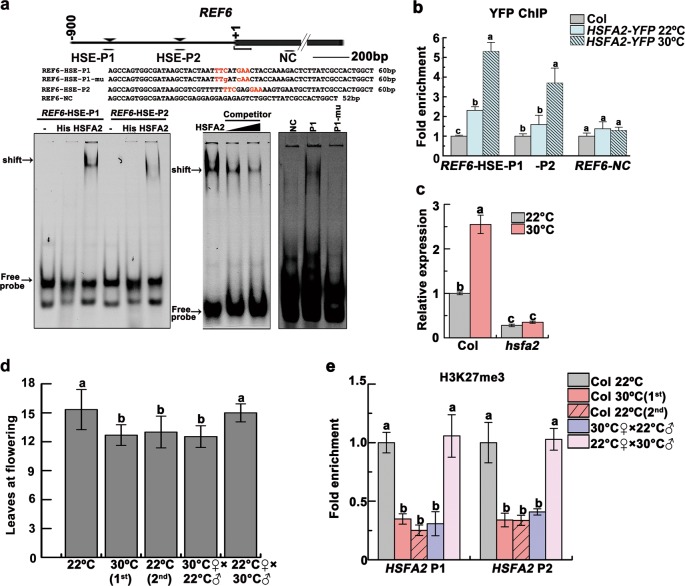
REF6 and HSFA2 form a regulatory loop that is maternally transmitted. **a** EMSA shows that His-HSFA2 protein but not His by itself specifically binds to the first HSE (nTTCnnGAAn motif) of *REF6* promoter in vitro. Arrows indicated the shifted bands. Excess unlabeled probe could outcompete the labeled probe (middle). A mutant HSE and a *REF6-NC* sequence were used as negative controls (right).The motif is highlighted in red in the probe. The region validated by ChIP-qPCR in **b** was marked by a bar. **b** ChIP-qPCR validation of HSFA2 occupancy at the *REF6* promoter region. The data were normalized to the corresponding input fraction. The *REF6-NC* locus was used as the negative-control locus. **c** *REF6* transcript level in 24-day-old Col and *hsfa2* grown at 22 °C and 30 °C. **d** The progeny derived from crosses (F1) of Col 30 °C♀×Col 22 °C♂ but not Col 22 °C♀×Col 30 °C♂flowered earlier. Flowering time of different lines was assessed by counting leaf numbers in bolting plants (*n* ≥ 15 for each line). **e** Lower H3K27me3 levels of *HSFA2* persisted in the progeny derived from Col 30 °C♀×Col 22 °C♂but not Col 22 °C♀×Col 30 °C♂. Data were shown as means ± SD from three replicates (**b**, **c**, **e**). Lowercase letters indicate statistical significance based on one-way (**b**, **d**, **e**) or two-way (**c**) ANOVA with Tukey’s HSD post hoc analysis (*P* < 0.05)

Interestingly, *REF6* and *BRM* also contains HSEs within their promoter regions (Fig. 5a; Supplementary information, Fig. S9a). We postulated that upregulation of *HSFA2* would thus in turn activate *REF6* and *BRM*. Indeed, *HSFA2* directly binds to one HSE within the *REF6* promoter, and to all three HSEs within the *BRM* promoter in vitro (Fig. 5a; Supplementary information, Fig. S9a). Further, HSFA2-YFP was enriched at the HSEs within the *REF6* and *BRM* promoters in heat-stressed plants (Fig. 5b; Supplementary information, Fig. S9b). The *hsfa2* knockout plants displayed reduced expression of these genes (Fig. 5c; Supplementary information, Fig. S9c). Together, these data strongly suggest a positive feedback loop, in which heat-induced activation of *HSFA2* triggers upregulation of *REF6* and *BRM*, which in turn upregulates *HSFA2*. Our data also suggest that progeny inherit upregulation of the *REF6/BRM*-*HSFA2* feedback loop as a determinant upstream node of the thermomemory.

To determine the parental contribution in transgenerational H3K27me3 demethylation, we made reciprocal crosses between 22 °C-grown Col and 30 °C-grown Col, and analyzed the flowering time of their progeny. The progeny derived from Col 30 °C♀×Col 22 °C♂flowered as early as the progeny of 30 °C-grown Col and accumulated higher levels of *FT* as well as *HSFA2*, *SGIP1* and *BRM* transcripts, while the progeny of Col 22 °C♀×Col 30 °C♂flowered at similar time to 22 °C-grown Col (Fig. 5d; Supplementary information, Fig. S9d). The lower H3K27me3 levels of *HSFA2* were also found in the progeny of Col 30 °C♀×Col 22 °C♂ (Fig. 5e). The results demonstrate that H3K27me3 demethylation is most likely transmitted maternally.

To further investigate the role of H3K27me3 demethylation in thermomemory, we evaluated the H3K27me3 levels and expression of a set of REF6 targets including *HSFA3* and *HSFA7a*.^27,28^ These REF6 targets displayed decreased H3K27me3 levels, but their expression levels were not upregulated transgenerationally (Supplementary information, Figs. S7b and 10a-c). We hypothesized that H3K27me3 demethylation alone may be insufficient to upregulate gene expression transgenerationally. We then investigated the expression of other reported HSFA2 target genes^26^ and observed transgenerational upregulation of 11/26 HSFA2 target genes (Supplementary information, Fig. S10d, e). Interestingly, similar to *HSFA2* itself, all of these thermomemory genes, except *Stress-inducible protein 1*(*Sti1*), showed heat-induced depletion of H3K27me3 that was generally transmitted to the progeny (Supplementary information, Fig. S10f). The biological importance of these thermomemory genes is worth further investigation. These data imply that heritable REF6/BRM module-mediated H3K27me3 demethylation and HSFA2 activation cooperate to maintain the transgenerational “ON state” of thermomemory genes including *HSFA2*.

### *HTT5* coordinates thermomemory phenotypes downstream of reduced tasiRNAs and the REF6-HSFA2 loop

**Fig. 6:**
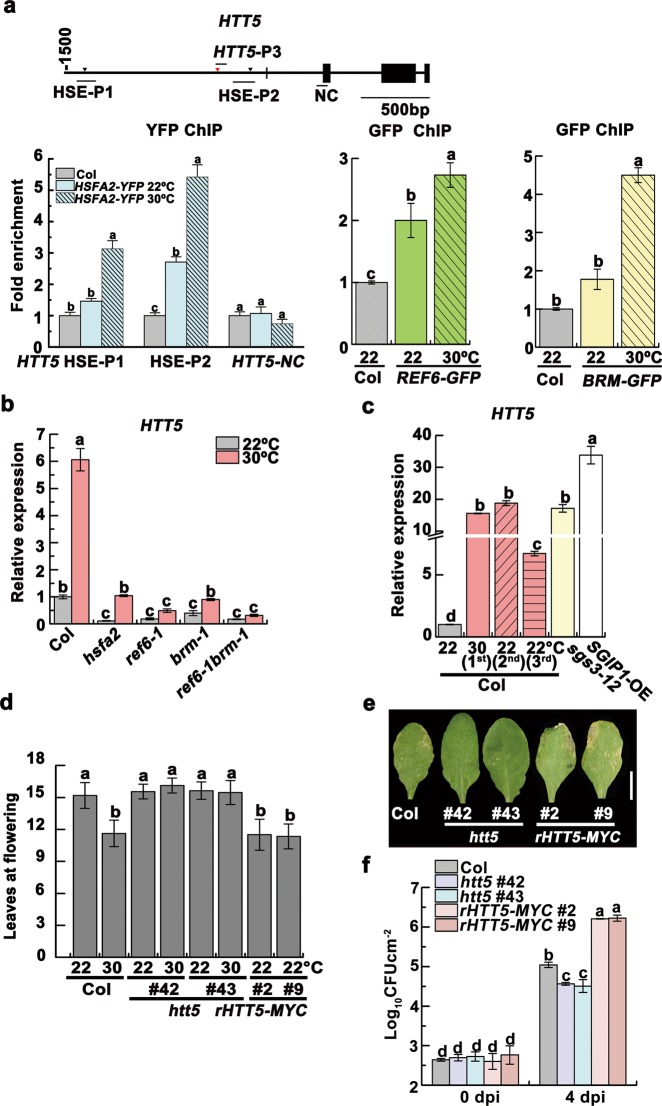
*HTT5* coordinates thermomemory phenotypes via reduced tasiRNAs and the REF6-HSFA2 loop. **a** ChIP-qPCR validation of HSFA2 (left), REF6 (middle) and BRM (right) occupancy at the *HTT5* loci. The analyzed regions were marked by bars. **b** *HTT5* transcript levels in 24-day-old Col, *hsfa2*, *ref6–1*, *brm-1* and *ref6–1brm-1* grown at 22 °C and 30 °C. **c** Heat-induced upregulation of *HTT5* could be transmitted to progeny, as detected by qRT-PCR. **d** Flowering times of indicated lines were assessed (*n* ≥ 15 for each line). **e** Disease symptoms at 4 dpi with *Pst* DC3000 (*avrRpt2*). Scale bar, 1 cm. **f** Bacterial growth was measured. Data are shown as means ± SD from three replicates (**a**, **b**, **c**, **f**). Lowercase letters indicate statistical significance based on one-way (**a**, **c**, **d**) or two-way (**b**, **f**) ANOVA with Tukey’s HSD post hoc analysis (*P* < 0.05)

**Fig. 7:**
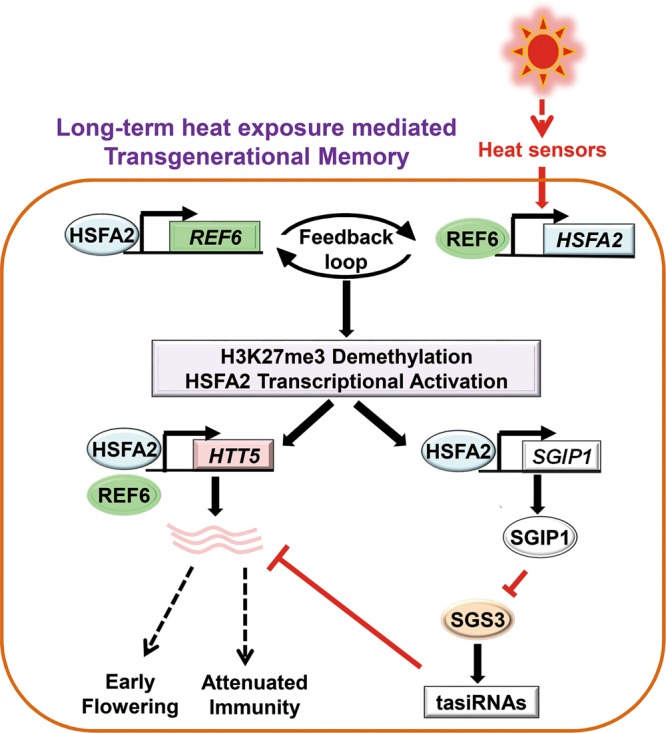
A proposed model depicting transgenerational thermomemory that is mediated by the H3K27me3 demethylation-HSFA2 regulatory loop in *Arabidopsis*. Long-term heat exposure activates plant heat sensor(s) that upregulates *HSFA2*, which targets and activates H3K27me3 demethylase REF6 and the chromatin remodeler BRM. REF6/BRM in turn triggers H3K27me3 demethylation to activate *HSFA2*. Thus, REF6 and HSFA2 form a heritable transcriptional feedback loop in heat responses and memory. This regulatory loop activates the E3 ligase, SGIP1, to trigger the transgenerational degradation of SGS3, leading to the suppression of tasiRNA biosynthesis. The REF6-HSFA2 loop and reduced tasiRNA levels converge to activate *HTT5*, which coordinates early flowering with decreased disease resistance in heat-stressed plants and progeny

To further uncover the tasiRNA target(s) that functions in the heat-induced memory of early flowering and immunity inhibition as described above, we analyzed the H3K27me3 status and expression of tasiRNA target genes. We found that the siR255 target *HTT5* is a co-target of HSFA2 and REF6/BRM, and its expression is regulated by H3K27me3 (Fig. 6a, b; Supplementary information, Fig. S11a-d). Further, heat-induced upregulation of *HTT5* was inherited by progeny (Fig. 6c). Importantly, the *htt5* mutant plants displayed neither heat-sensitive early flowering nor enhanced disease susceptibility to *Pst* DC3000 (*avrRpt2*) (Fig. 6d–f; Supplementary information, Fig. S12a, b). Further, we generated transgenic plants overexpressing a siR255-resistant version of *HTT5 (rHTT5-MYC*) and found that they flowered earlier with upregulated *FT* expression (Fig. 6d; Supplementary information, Fig. S12c, d). Compared to Col, the *rHTT5-MYC* lines were also more susceptible to *Pst* DC3000 (*avrRpt2*) (Fig. 6e, f). These results reveal that *HTT5*, the tasiRNA target, plays a dual role in growth and immunity: positively regulating reproductive development but negatively regulating immunity (Fig. 7). How HTT5 coordinates the heat-induced changes in development and immunity remains to be determined.

## Discussion

Here, we reveal that reduced H3K27me3 and tasiRNA biogenesis are necessary and sufficient to induce several transgenerational responses to heat stress, including early flowering, attenuated immunity, and PTGS release. We discovered an E3 ligase, SGIP1, which is upregulated by mild heat and mediates ubiquitination and degradation of SGS3 transgenerationally, and controls tasiRNA biogenesis. HSFA2 activates the expression of *SGIP1*, and *HSFA2* is activated by heat-induced demethylation of H3K27me3. Importantly, HSFA2 upregulates the expression of H3K27me3 demethylase REF6, establishing a positive feedback loop between H3K27me3 demethylation and HSFA2 upregulation that is critical to the thermomemory establishment and transmission to progeny, which further supports the transgenerational inheritance of gene activation at several target genes. In particular, heat exposure upregulates the tasiRNA target *HTT5* to promote early flowering but inhibit immunity, and the thermomemory phenotypes are transmitted to unstressed progeny (Fig. 7). Importantly, we reveal the complex epigenetic mechanisms that control transgenerational degradation of SGS3 and ensure the thermomemory phenotypes via the upregulation of the tasiRNA target *HTT5*. These findings provide a paradigm for understanding how environmental exposure can influence the evolutionary fitness of progeny in plants.

Whether and how histone modifications function in stress memory is a mystery. Recently, histone modifications were revealed as a conserved mechanism underlying long-term transmission of the silenced state, including H3K27me3 in *Drosophila* and H3K9 methylation in *Saccharomyces*.^29–32^ Interestingly, in *Caenorhabditis elegans*, heat stress-induced H3K9me3 demethylation and gene activation can persist for at least 14 generations.^33^ Most recently, the reduction of H3K9me3 and H3K27me3 was found associated with the transgenerational derepression of a silenced transgene in *C. elegans* exposed to the chemical Bisphenol A, dependent on demethylases.^34^ In *Arabidopsis*, prolonged exposure to cold results in the mitotically heritable but meiotically intransmissible silencing of *FLC* through the maintenance of H3K27me3.^35^ Cold-triggered and H3K27me3-mediated silencing is reset during each generation.^36^ Therefore, the meiotic ‘memory of heat’ observed in our study is distinct from the mitotic ‘memory of cold’. We found that H3K27me3 demethylation transmits thermomemory maternally. Similar maternal contribution was also observed in embryogenesis,^37^ and seed dormancy entrained by maternal temperature history in *Arabidopsis*.^38,39^ Interestingly, H3K27me3-maintained transgenerational silencing is also maternally inherited in *Drosophila*.^40^ Thus, H3K27me3 or H3K9me2/3 may be conserved heritable epigenetic marks that convey stress memories and adaptation in plants and animals.

Notably, we revealed that thermomemory has a biologically important impact on plant adaptation, given the early flowering of progeny grown at the normal temperature. Interestingly, heat stress could increase seed production in a later generation.^41^ These findings suggest that heat exposure promotes species survival by enhancing reproduction. However, we found that unstressed progeny displayed attenuated disease resistance, suggesting that heat increases progeny adaptation at the cost of defense. These findings are consistent with an antagonistic relationship between plant immunity and plant growth and development. Warm ambient temperatures induce early flowering in many other plants, including rice. It would be of interest to determine whether the same epigenetic mechanism and SGS3 degradation are utilized by other plants to execute heat responses and thermomemory. Furthermore, how *HTT5* coordinates the heat-induced changes in development and immunity is of interest. We observed that these thermomemory phenotypes mostly lasted for 2 generations. This ensures plants to reset responses to ever changing environments.

We propose that H3K27me3 demethylation and HSFA2 activation might be key effectors downstream of heat sensing in plants. How sense heat is still an important topic to be addressed. It is difficult to define the primary heat sensor(s), given that heat simultaneously poses a stress to almost all macromolecules and all organelles in cells. Although several putative heat sensors have been proposed, including a cyclic nucleotide gated calcium channel (CNGC), two unfolded protein sensors, and H2A.Z-containing nucleosomes,^3^ it is unclear whether these potential sensors-mediated mechanisms are also responsible for the H3K27me3 demethylation-mediated thermomemory. It is possible that plants sense heat through different organelles during different phases of the response. Systematic screening for factors regulating transgenerational thermomemory will shed light on this issue, and might provide new knowledge and technology to improve thermotolerance in crops.

## Materials and methods

### Plant materials and growth conditions

All genetic stocks used in this paper are in Columbia background. *sgs3–12*,^19^ *p35S::SGS3-GFP*,^10^ *hsfa2*,^42^ *pHSFA2::HSFA2-YFP*,^43^ *brm-1*, *ref6–1*, *ref6–1brm-1*, *ref6–1 pREF6::REF6-GFP*, *brm-1 ProBRM::BRM-GFP*^27^ and *MIM480(+)*^8^ were described previously. The *sgip1* mutant (*SALK_017126C*) was obtained from the *Arabidopsis* Biological Resource Center. Homozygous T-DNA insertion mutants were identified by PCR-based genotyping. The progeny of Col 30 °C♀×Col 22 °C♂and Col 22 °C♀×Col 22 °C♂were derived from reciprocal crosses between 22 °C-grown Col and 30 °C-grown Col. After crossing, the parent plants were all kept at 22 °C and the seeds were harvested for further analysis.

The seeds were plated on 1/2 Murashige and Skoog (MS) agar medium and stratified at 4 °C in darkness for 4 day, and then moved to a greenhouse and germinated at 22 °C for 1 week. The seedlings were transplanted to the soil in plastic pots and grown at 22 °C under long-day conditions (16-h light/8-h dark) and 50–70% relative humidity as previously described.^10^ For heat treatment, 10-day-old seedlings grown at 22 °C were transferred into a growth chamber and grown at 30 °C for 2 weeks with 16-h light/8-h dark cycles and 50–70% relative humidity as previously described.^10^ All flowering plants were moved back to 22 °C to produce seeds (Supplementary information, Figure S1a). All air-dried seeds were collected and kept at 4 °C.

### Flowering time assessment

Wild-type and mutant plants were grown side by side in soil at 22 °C or 30 °C. To compare flowering times among different genotypes, total rosette and cauline leaves were counted when the plants flowered. At least fifteen plants were counted for each line and the analysis was repeated three times independently.

### Plasmid constructs and plant transformation

The full-length genomic sequences of *SGIP1* and *HSFA2* were cloned into pCambia1300 to generate *p35S::SGIP1* and *p35S::HSFA2–4MYC*. The *siR480(+)*/*siR255*-resistant version of *HTT5* was also cloned into pCambia1300 to generate *p35S::rHTT5–4MYC*. Two sgRNAs were designed and fused to pCAMBIA1300-*pYAO*:*Cas9*^44^ to delete a region (+441 ~ +544 from the first codon) containing CTCTGYTY motifs in the intron of *HSFA2*. Two sgRNAs targeting the exon of *HTT5* were fused to pCAMBIA1300-*pYAO*:*Cas9* to mutate *HTT5*. All the constructs were introduced into *Agrobacterium tumefaciens* strain GV3101 to transform Col plants, leading to the generation of more than 15 transgenic plants. For selection of transgenic plants, the seeds were sterilized and germinated on 1/2 MS agar medium containing 25 mg/L hygromycin. All primer sequences used for cloning can be found in Supplementary information, Table S2.

### RNA analysis

Total RNAs were extracted using TRIzol reagent from the fourth and fifth true leaves from 24-day-old plants. RNA was quantified using NanoDrop, and approximately 1 µg total RNA was converted into cDNA using HiScript Q RT SuperMix (Vazyme, R123–01) for quantitative real-time PCR (qRT-PCR) according to the manufacturer instructions. 2 μL of the resulting 20 μL cDNAs was used for qRT-PCR analyses with the primers listed in Supplementary Table 2. The qRT-PCR reactions were performed using a CFX96 Real-time PCR Instrument (Bio-Rad) with SYBR^®^ Premix Ex Taq™ (Takara, RR420A). Three biological repeats were analyzed. qRT-PCR data were normalized to the *ACTIN2* signals.

To analyze small RNAs, 20–30 μg of total RNAs was separated by electrophoresis on 15% polyacrylamide/7 M urea denaturing gels and transferred to nylon membrane (GE Health) for 1 h at 3.3 mA/cm^2^ using semi-dry transfer unit (Bio-Rad). After cross-linking by UV irradiation, the membrane was hybridized in PerfectHyb^TM^ Plus hybridization buffer (Sigma-Aldrich, H7033) with biotin-labeled DNA probes complementary to the siRNA or miRNA sequences at 42 °C overnight. Detection of immobilized nucleic acids was performed using Nucleic Acid Detection Module Kit (Thermo Fisher, 89880). After stripping the probes, the same blots were reprobed with U6 probe that serves as a loading control.

### Chromatin immunoprecipitation (ChIP)

ChIP experiments were performed as described.^45^ Briefly, 2 g four-week-old 22 °C- or 30 °C-grown seedlings were harvested and cross-linked with 1% formaldehyde for 10 min under vacuum. For validation of REF6 binding, 0.5 g flowers from 22 °C- or 30 °C-grown plants were used. Cross-linking was stopped by adding glycine. The seedlings were rinsed with water, and then ground into fine powder in liquid nitrogen. Chromatin was isolated and sheared into 200–500 bp fragments by sonication. The sonicated chromatin was incubated with antibodies against GFP (Abcam, ab290) or H3K27me3 (Millipore, 07–449) overnight at 4 °C. Immunocomplexes were precipitated, washed and eluted and the cross-links were reversed at 65 °C overnight. Precipitated DNA was then recovered with the Dr.GenTLE^TM^ precipitation carrier (Takara, 9094) according to the manufacturer’s instructions. Background control ChIP was performed using intergenic regions without H3K27me3 mark to determine the ChIP specificity. ChIP-qPCR was performed with three replicates and relative fold enrichments over the background were shown. ChIP experiments were performed independently at least three times with similar results. Sequences of the primers used for ChIP-qPCR are listed in Supplementary information, Table S2.

### Electrophoretic mobility shift assays (EMSA)

The full-length coding sequence of *HSFA2* was cloned into pET32a and expressed in *E. coli* Rosetta (DE3). The His-tagged HSFA2 fusion protein was purified. GST and GST-REF6C fusion proteins were expressed and purified as described.^28^ DNA fragments were end-labeled with Cy5. 200 ng His or His-HSFA2 protein was supplemented with 10 ng Cy5-labeled DNAs and incubated in the binding buffer (10 mM Tris-HCl, pH 7.6, 25 mM KCl, 2.5 mM MgCl_2_, 1 mM EDTA, 1 mM DTT, and 10% glycerol) at 25 °C for 30 min. For REF6 binding activity detection, 200 ng protein was mixed with 10 ng Cy5-labeled DNAs in the binding buffer (25 mM Tris-HCl, 100 mM NaCl, 2.5 mM MgCl_2_, 0.1% CA-630, 10% glycerol, 1 µM ZnSO_4_, and 1 mM DTT, pH 8.0) and incubated on ice for 1 h. For competition assays, 50- or 100-fold non-labeled competitor DNA was added in the reaction 10 min before the addition of the cy5-labeled probe. The reaction mixtures were resolved on 4.5% native polyacrylamide gels in 0.5× TBE at 100 V for 1–2 h on ice in dark. Gels were directly scanned using FLA 2000 (Fuji).

### Protein analysis

The anti-SGS3 and anti-SGIP1 polyclonal antibodies were developed by ABclonal^®^ Technology (Wuhan, China). The SGS3ΔN (290–625aa) and full-length CDS of SGIP1 were cloned into pET32a and expressed in Rosetta (DE3). The His-tagged SGS3ΔN and SGIP1 proteins were purified and used as antigens to raise polyclonal antibodies in rabbit, respectively.

To extract total proteins, 0.1 g fifth true leaves from 24-day-old plants were ground into fine power in liquid nitrogen, homogenized in the protein extraction buffer (150 mM Tris-HCl, pH 7.5, 6 M urea, 2% SDS, and 5% β-mercaptoethanol), boiled for 5 min and then centrifuged at 13,200 rpm for 10 min at 4 °C to remove debris. Proteins were resolved on a 4–20% Biofuraw^TM^ Precast Gel (Tanon) by electrophoresis and transferred to PVDF membranes at room temperature for 10–20 min at 25 V using Trans-Blot^®^ Turbo^TM^ Blotting System (BIO-RAD). Signals were visualized with High-sig ECL Western Blotting Substrate (Tanon, 180–501), and images were captured using the Tanon-5200 Chemiluminescent imaging system (Tanon). Quantification of protein signal was performed using Image J software. Antibodies against the following proteins were used: HSFA2 (1:800 dilution),^42^ MYC (MILLIPORE, 05–724; 1:2,000 dilution), FLAG (Sigma, F1804; 1:2,000 dilution), SGS3 (1:1,000 dilution), SGIP1 (1:1,000 dilution), GFP (Abcam, ab290; 1:2,000 dilution), ACTIN (CMCTAG, AT0004; 1:2,000 dilution). ACTIN was used as a loading control.

The co-immunoprecipitatin (Co-IP) procedure was described previously.^46^ Harvested samples were grounded in liquid nitrogen, homogenized in the lysis buffer (50 mM Tris-HCl, pH 7.5, 150 mM NaCl, 1 mM EDTA, 10% glycerol, 1% Triton X-100, 1 mM PMSF and 1× protease inhibitor cocktail), incubated at 4 °C for 30 min, and centrifuged at 10,000 g for 15 min. The supernatant was mixed with prewashed anti-FLAG M2 magnetic beads (Sigma-Aldrich, M8823), incubated at 4 °C for 2 h and washed three times with lysis buffer. The bound proteins were eluted from the affinity beads by boiling for 5 min in 40 μL 2× SDS loading buffer and analyzed by immunoblot.

For cell-free protein degradation assay, total proteins were extracted from 2-week-old seedlings with the extraction buffer (50 mM Tris-MES, pH 8.0, 0.5 M sucrose, 1 mM MgCl_2_, 10 mM EDTA, 5 mM DTT, 1 mM phenylmethylsulfonyl fluoride, and 1× protease inhibitor cocktail) as described previously.^47^ Samples were incubated at 22 °C or 30 °C in the presence of 100 μM CHX (Sigma-Aldrich, C4859) or MG132 (Sigma-Aldrich, C2211) for various time periods. All protein immunodetection experiments were performed independently three times with similar results.

### Yeast two-hybrid assay

The full-length CDS of SGS3 and SGIP1 and domain deletion mutants SGS3Δ220, SGS3ΔZF, SGS3ΔXS, SGS3ΔCC, SGIP1ΔF, and SGIP1ΔFAID were amplified and subcloned into the pDONR207 vector by BP reaction. The inserts were then fused to the GAL4 activation domain in pDEST-GADT7 or GAL4 DNA binding domain in pDEST-GBKT7 by LR reaction. The combinations of bait and prey constructs were co-transformed into yeast strain AH109 and selected on dropout medium without leucine and tryptophan. To assay protein-protein interactions, clones were grown on dropout medium without leucine, histidine and tryptophan containing 2 mM 3-aminotriazole (Sigma-Aldrich, A8056).

### Protein ubiquitination assays

The in vitro ubiquitination assay was carried out as described previously.^46^ SGS3 protein was transiently expressed in *N. benthamiana* and purified by immunopreciptation using an anti-SGS3 antibody. For the in vitro ubiquitination assay, the reaction mixture (30 μL) containing 50 ng AtE1, 200 ng AtUBC10, 1 μg ubiquitin, 1 μg purified SGIP1 fused with the MBP tag, and 100 ng SGS3 protein was incubated for 1.5 h at 30 °C with agitation. The reaction was stopped by the addition of 4× protein loading buffer and boiling for 5 min. Samples were separated on a 7.5% SDS-PAGE gel and the ubiquitinated SGS3 was detected by immunoblotting with an anti-SGS3 antibody. The mutated SGIP1 protein (SGIP1^ΔFbox^) was used as a negative control.

In vivo ubiquitination assay by agroinfiltration was performed following the protocol described previously.^48^ Coinfiltration expression of SGIP1 and SGS3 was performed in *N. benthamiana* leaves. MG132 was injected into the leaf tissues 12 h before harvest. Total proteins were extracted from leaf tissues and analyzed by immunoblotting with the anti-SGS3, anti-FLAG and anti-GFP antibodies. GFP was co-infiltrated as an internal control and ACTIN was used as a loading control.

### Protoplast isolation and transfection

The plasmids PA7-SGS3-GFP and PA7-SGIP1-RFP were constructed and purified with HiSpeed Plasmid Midi Kit (QIAGEN, 12643). The mesophyll protoplasts were isolated from the leaves of 3-week-old Col using the Tape-*Arabidopsis* Sandwich procedure.^49^ The protoplasts were transfected with 20 μg purified DNA using PEG solution (40% (w/v) PEG, 0.1 M CaCl_2_, and 0.2 M mannitol). After incubation at room temperature for 8 mins, W5 buffer (154 mM NaCl, 125 mM CaCl_2_, 5 mM KCl, 5 mM glucose, and 2 mM MES, pH 5.7) was added to stop the transfection. The protoplasts were collected and resuspended with W5 buffer, and then transferred into 6-well plates and incubated overnight. YFP and RFP signals were observed with an Olympus FV1000 confocal laser scanning microscope.

### Bacterial disease assays and SA measurement

Bacterial disease assays were performed as described.^50^ *Pseudomonas syringae* pv. *Tomato* (*Pst*) carrying effector *avrRpt2* [*Pst* DC3000(*avrRpt2*)] was used in this study. Two-week-old plants were shifted from 22 °C to 30 °C or continuously grown at 22 °C for another 1 week before infection. Bacteria were collected by centrifugation and resuspended in sterile water to OD600 = 0.2 (~1 × 10^8^ cfu/ml). Bacterial suspension was further diluted to OD600 = 0.0002 before syringe-infiltration on the fifth true leaves of plants. Water infiltration was performed as mock inoculation. At 0 and 4 days post inoculation (dpi), leaf discs from ten leaves were collected from each genotype, surface-sterilized with 75% ethanol, rinsed in sterile water, and ground in sterile water for bacteria extraction. Bacterial titer was determined by plating a serial dilution of the suspension onto Luria-Marine plates with 100 μg/ml Rifampicin and 50 μg/ml kanamycin and incubated for 2 days at 28 °C, with three biological repeats. Experiments were repeated independently at least three times.

SA was extracted and quantified as previously described.^51^ 0.1 g leaf tissues from plants syringe-infiltrated with water or *Pst* DC3000(*avrRpt2*) were ground for SA extraction, with four biological replicates of each genotype. Contents of SA were determined by GC-MS using a labeled internal standard.

### Luciferase biomolecular complementation assay

SGS3 and SGIP1 were fused to N- or C-terminus of firefly luciferase and transformed to *Agrobacterium* strain GV3101. Overnight cultures of *Agrobacteria* were collected by centrifugation, resuspended in MES buffer (10 mM MgCl_2_,10 mM MES, 150 μM AS, pH 5.6), mixed with GV3101 expressing P19 to OD600 of 0.5, and incubated at room temperature for 3–4 h before infiltration. Healthy leaves of 3-week-old *N. benthamiana* were syringe-infiltrated. 2 days later, the LUC activity was assayed with Luciferase Assay Systems (Promega, E1501) and images were captured using the Tanon-5200 Chemiluminescent imaging system (Tanon).

### Statistical analysis

For qPCR analyses, ChIP, flowering time assessment, disease assays and SA level detection, significant differences between samples/lines and the corresponding controls were analyzed using a Student’s *t*-test for pairwise comparisons, one-way or two-way ANOVA analysis with Tukey’s multiple comparison test as specified in the legends. Samples sharing lowercase letters are not significantly different.

## Supplementary information

Supplementary information, Figure S1

Supplementary information, Figure S2

Supplementary information, Figure S3

Supplementary information, Figure S4

Supplementary information, Figure S5

Supplementary information, Figure S6

Supplementary information, Figure S7

Supplementary information, Figure S8

Supplementary information, Figure S9

Supplementary information, Figure S10

Supplementary information, Figure S11

Supplementary information, Figure S12

Supplementary information, Table S1

Supplementary information, Table S2

## References

[1] Pauli H (2012). Recent plant diversity changes on Europe’s mountain summits. Science.

[2] Lobell DB, Schlenker W, Costa-Roberts J (2011). Climate trends and global crop production since 1980. Science.

[3] Liu J, Feng L, Li J, He Z (2015). Genetic and epigenetic control of plant heat responses. Front. Plant Sci..

[4] Tittel-Elmer M (2010). Stress-induced activation of heterochromatic transcription. PLoS Genet..

[5] Pecinka A (2010). Epigenetic regulation of repetitive elements is attenuated by prolonged heat stress in *Arabidopsis*. Plant Cell.

[6] Ito H (2011). An siRNA pathway prevents transgenerational retrotransposition in plants subjected to stress. Nature.

[7] Lang-Mladek C (2010). Transgenerational inheritance and resetting of stress-induced loss of epigenetic gene silencing in *Arabidopsis*. Mol. Plant.

[8] Li S (2014). *HEAT-INDUCED TAS1 TARGET1* mediates thermotolerance via HEAT STRESS TRANSCRIPTION FACTOR A1a-directed pathways in *Arabidopsis*. Plant Cell.

[9] Yu X (2013). Global analysis of cis-natural antisense transcripts and their heat-responsive nat-siRNAs in *Brassica rapa*. BMC Plant Biol..

[10] Zhong SH (2013). Warm temperatures induce transgenerational epigenetic release of RNA silencing by inhibiting siRNA biogenesis in. Arab. Proc. Natl Acad. Sci. USA.

[11] Lamke J, Baurle I (2017). Epigenetic and chromatin-based mechanisms in environmental stress adaptation and stress memory in plants. Genome Biol..

[12] Iwasaki M, Paszkowski J (2014). Identification of genes preventing transgenerational transmission of stress-induced epigenetic states. Proc. Natl Acad. Sci. USA.

[13] Migicovsky Z, Yao Y, Kovalchuk I (2014). Transgenerational phenotypic and epigenetic changes in response to heat stress in *Arabidopsis thaliana*. Plant Signal. Behav..

[14] Fukunaga R, Doudna JA (2009). dsRNA with 5' overhangs contributes to endogenous and antiviral RNA silencing pathways in plants. EMBO J..

[15] Kumar SV (2012). Transcription factor PIF4 controls the thermosensory activation of flowering. Nature.

[16] Huot B (2017). Dual impact of elevated temperature on plant defence and bacterial virulence in *Arabidopsis*. Nat. Commun..

[17] Gangappa SN, Berriri S, Kumar SV (2017). PIF4 coordinates thermosensory growth and immunity in. Arab. Curr. Biol..

[18] Allen E, Xie Z, Gustafson AM, Carrington JC (2005). microRNA-directed phasing during *trans*-acting siRNA biogenesis in plants. Cell.

[19] Peragine A, Yoshikawa M, Wu G, Albrecht HL, Poethig RS (2004). *SGS3* and *SGS2*/*SDE1*/*RDR6* are required for juvenile development and the production of *trans*-acting siRNAs in. Arab. Genes Dev..

[20] Karasov TL, Chae E, Herman JJ, B J (2017). Mechanisms to mitigate the trade-off between growth and defense. Plant Cell.

[21] Deng Y (2017). Epigenetic regulation of antagonistic receptors confers rice blast resistance with yield balance. Science.

[22] Mazzucotelli E (2006). The E3 ubiquitin ligase gene family in plants: regulation by degradation. Curr. Genom..

[23] Gould PD (2006). The molecular basis of temperature compensation in the *Arabidopsis* circadian clock. Plant Cell.

[24] Howell MD (2007). Genome-wide analysis of the RNA-DEPENDENT RNA POLYMERASE6/DICER-LIKE4 pathway in *Arabidopsis* reveals dependency on miRNA- and tasiRNA-directed targeting. Plant Cell.

[25] Rajagopalan R, Vaucheret H, Trejo J, Bartel DP (2006). A diverse and evolutionarily fluid set of microRNAs in *Arabidopsis thaliana*. Genes Dev..

[26] Nishizawa A (2006). *Arabidopsis* heat shock transcription factor A2 as a key regulator in response to several types of environmental stress. Plant J..

[27] Li C (2016). Concerted genomic targeting of H3K27 demethylase REF6 and chromatin-remodeling ATPase BRM in *Arabidopsis*. Nat. Genet..

[28] Cui X (2016). REF6 recognizes a specific DNA sequence to demethylate H3K27me3 and regulate organ boundary formation in *Arabidopsis*. Nat. Genet..

[29] Wang X, Moazed D (2017). DNA sequence-dependent epigenetic inheritance of gene silencing and histone H3K9 methylation. Science.

[30] Laprell F, Finkl K, Muller J (2017). Propagation of Polycomb-repressed chromatin requires sequence-specific recruitment to DNA. Science.

[31] Coleman RT, Struhl G (2017). Causal role for inheritance of H3K27me3 in maintaining the OFF state of a *Drosophila* HOX gene. Science.

[32] Yu R, Wang X, Moazed D (2018). Epigenetic inheritance mediated by coupling of RNAi and histone H3K9 methylation. Nature.

[33] Klosin A, Casas E, Hidalgo-Carcedo C, Vavouri T, Lehner B (2017). Transgenerational transmission of environmental information in *C. elegans*. Science.

[34] Camacho J (2018). The memory of environmental chemical exposure in *C. elegans* is dependent on the jumonji demethylases *jmjd-2* and *jmjd-3/utx-1*. Cell Rep..

[35] Yang H (2017). Distinct phases of Polycomb silencing to hold epigenetic memory of cold in *Arabidopsis*. Science.

[36] Tao Z (2017). Embryonic epigenetic reprogramming by a pioneer transcription factor in plants. Nature.

[37] Autran D (2011). Maternal epigenetic pathways control parental contributions to Arabidopsis early embryogenesis. Cell.

[38] Chen M (2014). Maternal temperature history activates Flowering Locus T in fruits to control progeny dormancy according to time of year. Proc. Natl Acad. Sci. USA.

[39] Chen M, Penfield S (2018). Feedback regulation of COOLAIR expression controls seed dormancy and flowering time. Science.

[40] Zenk F (2017). Germ line-inherited H3K27me3 restricts enhancer function during maternal-to-zygotic transition. Science.

[41] Whittle CA, Otto SP, Johnston MO, Krochko JE (2009). Adaptive epigenetic memory of ancestral temperature regime in *Arabidopsis thaliana*. Botany.

[42] Liu HC, Liao HT, Charng YY (2011). The role of class A1 heat shock factors (HSFA1s) in response to heat and other stresses in *Arabidopsis*. Plant Cell Environ..

[43] Lamke J, Brzezinka K, Altmann S, Baurle I (2016). A hit-and-run heat shock factor governs sustained histone methylation and transcriptional stress memory. EMBO J..

[44] Yan L (2015). High-efficiency genome editing in *Arabidopsis* using *YAO* promoter-driven CRISPR/Cas9 system. Mol. Plant.

[45] Wang H (2016). *Arabidopsis* flower and embryo developmental genes are repressed in seedlings by different combinations of polycomb group proteins in association with distinct sets of cis-regulatory elements. PLoS Genet..

[46] You Q (2016). An E3 ubiquitin ligase-BAG protein module controls plant innate immunity and broad-spectrum disease resistance. Cell Host Microbe.

[47] Zhang H (2015). The RING finger ubiquitin E3 ligase SDIR1 targets SDIR1-INTERACTING PROTEIN1 for degradation to modulate the salt stress response and ABA signaling in *Arabidopsis*. Plant Cell.

[48] Liu L, Zhao Q, Xie Q (2012). In vivo ubiquitination assay by agroinfiltration. Methods Mol. Biol..

[49] Wu FH (2009). Tape-*Arabidopsis* Sandwich - a simpler *Arabidopsis* protoplast isolation method. Plant Methods.

[50] Xin XF (2016). Bacteria establish an aqueous living space in plants crucial for virulence. Nature.

[51] Yin X (2018). Rice copine genes *OsBON1* and *OsBON3* function as suppressors of broad-spectrum disease resistance. Plant Biotechnol. J..

