# Supplementary material for: An H3K27me3 demethylase-HSFA2 regulatory loop orchestrates transgenerational thermomemory in Arabidopsis
Source: Cell Res. 2019 Feb 18;29(5):379–90. doi: 10.1038/s41422-019-0145-8 (PMC6796840; doi:10.1038/s41422-019-0145-8)
Supplement: Supplementary file 1 — Supplementary information, Figure S1 [file 41422_2019_145_MOESM1_ESM.pdf]

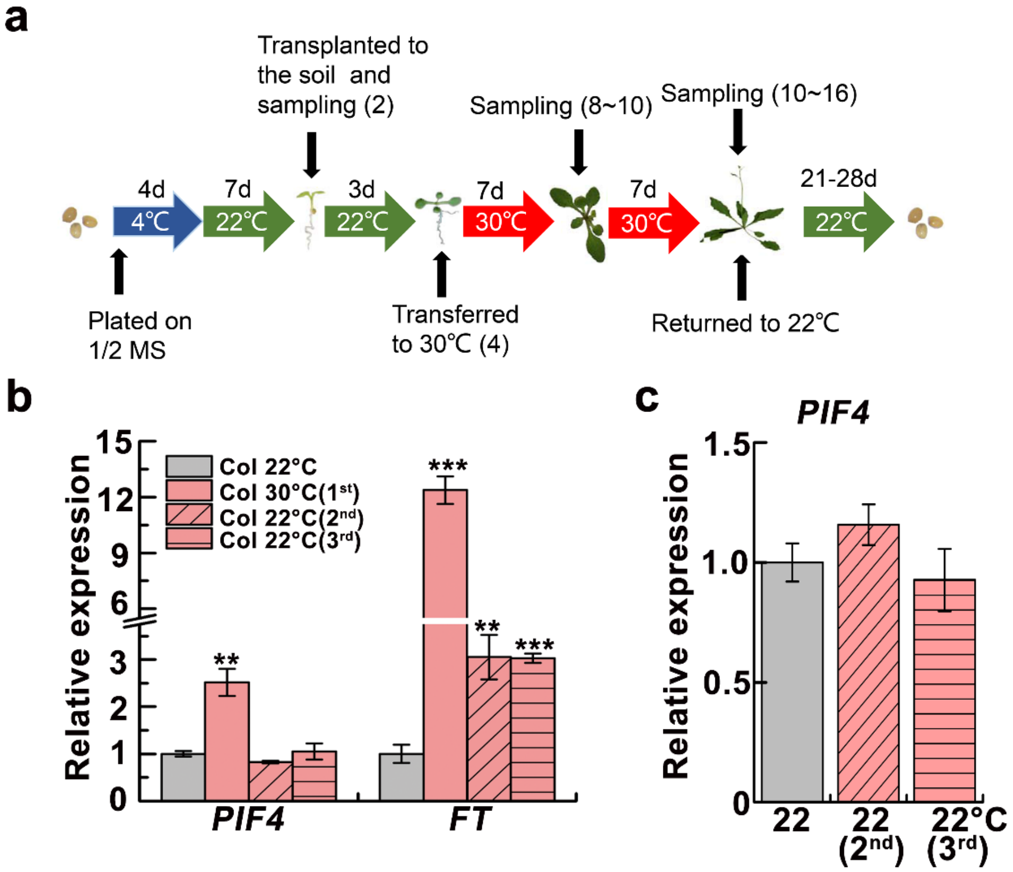
a
Transplanted to the soil and sampling (2)
Sampling (8~10)
Sampling (10~16)
4d
4℃
7d
22℃
3d
22℃
7d
30℃
7d
30℃
21-28d
22℃
Plated on 1/2 MS
Transferred to 30℃ (4)
Returned to 22℃
b
Relative expression
Col 22°C
Col 30°C(1st)
Col 22°C(2nd)
Col 22°C(3rd)
PIF4
FT
c
PIF4
Relative expression
22
22 (2nd)
22°C (3rd)

Supplementary Figure 1. Experimental design for prolonged heat treatment and *PIF4* is not involved in the transgenerational thermomemory.

a Experimental design for prolonged heat treatment. The seeds were plated on 1/2 Murashige and Skoog (MS) agar medium and stratified at 4 ℃ in darkness for 4 d, and then moved to a green house and germinated at 22 ℃ for 7 d under long-day conditions (16-h light/8-h dark) and 50–70% relative humidity as previously described.[10] The two-leaf stage seedlings were transplanted to the soil in plastic pots and grown at 22 ℃ for additional 3 d for acclimation. The 10-day-old four-leaf stage seedlings were transferred into a growth chamber and grown at 30 ℃ for 14 d with 16-h light/8-h dark cycles and 50–70% relative humidity. All flowering plants were moved back to 22 ℃ to produce seeds. All seeds were air-dried and kept at 4 ℃. RNA and protein samples were collected from 17-day-old or 24-day-old seedlings as indicated in each figure legends. The numbers in brackets indicate the leaf numbers at each stage.

b *PIF4* and *FT* transcript levels as normalized to the *ACTIN2* signals in 24-day-old seedlings. The average values (± s.d., n = 3) were shown.

c Analysis of the relative transcript levels of *PIF4* in 7-day-old 22 °C-grown Col, 2nd and 3rd generation offspring of 30 °C-grown Col. Asterisks indicate significant difference (Student's *t* test; $*p < 0.05$, $**p < 0.01$, $***p < 0.001$) (**b**, **c**).
