# Supplementary material for: An H3K27me3 demethylase-HSFA2 regulatory loop orchestrates transgenerational thermomemory in Arabidopsis
Source: Cell Res. 2019 Feb 18;29(5):379–90. doi: 10.1038/s41422-019-0145-8 (PMC6796840; doi:10.1038/s41422-019-0145-8)
Supplement: Supplementary file 2 — Supplementary information, Figure S2 [file 41422_2019_145_MOESM2_ESM.pdf]

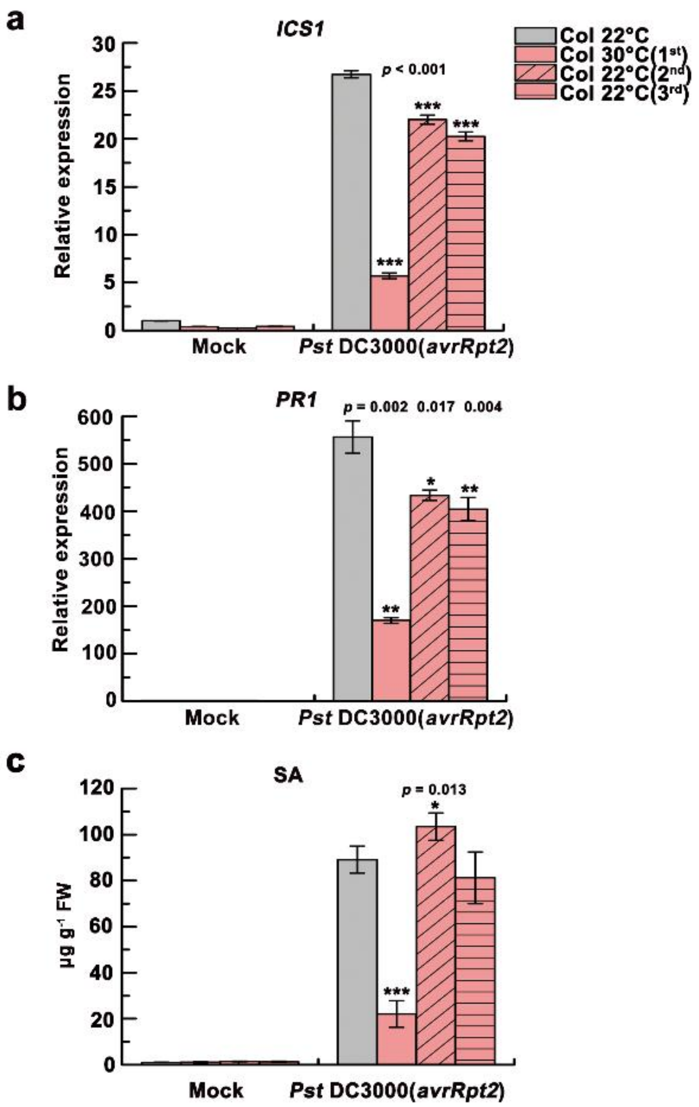
a
ICS1
Col 22°C
Col 30°C(1st)
Col 22°C(2nd)
Col 22°C(3rd)
Relative expression
30
25
20
15
10
5
0
p < 0.001
***
***
***
Mock
Pst DC3000(avrRpt2)
b
PR1
p = 0.002 0.017 0.004
600
500
400
300
200
100
0
Relative expression
**
*
**
Mock
Pst DC3000(avrRpt2)
c
SA
p = 0.013
120
100
80
60
40
20
0
μg g-1 FW
***
*
Mock
Pst DC3000(avrRpt2)

Supplementary Figure 2. The heat-induced memory of attenuated immunity is likely independent of SA production.

a,**b** The expression of SA marker genes *ICS1* (**a**) and *PR1* (**b**) 15 h after syringe-infiltration with water (mock) or *Pst* DC3000(*avrRpt2*) (1×10^{7} cfu ml^{-1}). qPCR data were normalized to the *ACTIN2* signals and shown as means ± s.d. from three technical replicates.

c Total SA quantification with water (mock) or *Pst* DC3000(*avrRpt2*) (1×10^{7} cfu ml^{-1}) inoculation. SA was quantified using LC and normalized by fresh weight (FW) (g) of samples. Values are means ± s.d. from four technical replicates.

Asterisks indicate significant difference from the 22 °C-grown Col (Student's *t* test; *$p < 0.05$, **$p < 0.01$, ***$p < 0.001$) (**a-c**).
