# Supplementary material for: An H3K27me3 demethylase-HSFA2 regulatory loop orchestrates transgenerational thermomemory in Arabidopsis
Source: Cell Res. 2019 Feb 18;29(5):379–90. doi: 10.1038/s41422-019-0145-8 (PMC6796840; doi:10.1038/s41422-019-0145-8)
Supplement: Supplementary file 3 — Supplementary information, Figure S3 [file 41422_2019_145_MOESM3_ESM.pdf]

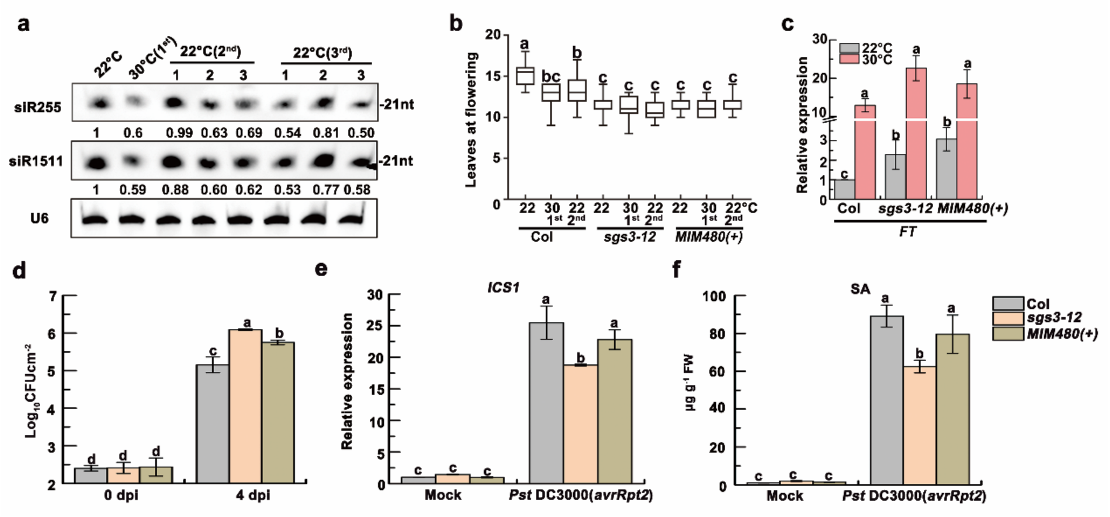
a
22°C
30°C(1st)
22°C(2nd)
22°C(3rd)
siR255
-21nt
1 0.6 0.99 0.63 0.69 0.54 0.81 0.50
siR1511
1 0.59 0.88 0.60 0.62 0.53 0.77 0.58
U6
b
Leaves at flowering
Col
sgs3-12
MIM480(+)
c
Relative expression
22°C
30°C
FT
d
Log10CFUcm-2
0 dpl
4 dpl
e
ICS1
Relative expression
Mock
Pst DC3000(avrRpt2)
f
SA
µg g-1 FW
Col
sgs3-12
MIM480(+)

Supplementary Figure 3. The heat-induced memory of early flowering and attenuated immunity is dependent on SGS3-mediated biogenesis of tasiRNAs.

a RNA blot of siR255 and siR1511 in three representative individuals of 2nd and 3rd progeny were analyzed, with relative levels shown under lanes. All progeny plants but plant 1 in 22 °C (2nd) showed early flowering. U6 served as a loading control.

b Box plots of flowering times of Col, *sgs3-12* and *MIM480(+)* plants of the indicated generations grown at 22 °C or 30 °C (n ≥ 15 for each line).

c Analysis of *FT* transcript levels in 22 °C or 30 °C-grown Col, *sgs3-12* and *MIM480(+)* plants.

d Bacteria grew more in *sgs3-12* and *MIM480(+)* plants compared with wild-type Col.

e,**f** Analysis of *ICS1* transcript levels (**e**) and SA levels (**f**) in Col, *sgs3-12* and *MIM480(+)* plants 15 h after syringe-infiltration with water or *Pst* DC3000(*avrRpt2*) (1×10^7 cfu ml^{-1}). qRT-PCR data were normalized to the *ACTIN2* signals and shown as means ± s.d. from three replicates (**c**, **e**). SA was quantified using LC and normalized by fresh weight (FW) (g) of samples. Values are means ± s.d. from four replicates (**f**). Figure **S2c** and Figure **S3f** shared the same wild-type control. Significant difference was determined by two-way (**b-f**) ANOVA with Tukey's HSD post hoc test (significance set at $p < 0.05$).
