# Supplementary material for: An H3K27me3 demethylase-HSFA2 regulatory loop orchestrates transgenerational thermomemory in Arabidopsis
Source: Cell Res. 2019 Feb 18;29(5):379–90. doi: 10.1038/s41422-019-0145-8 (PMC6796840; doi:10.1038/s41422-019-0145-8)
Supplement: Supplementary file 4 — Supplementary information, Figure S4 [file 41422_2019_145_MOESM4_ESM.pdf]

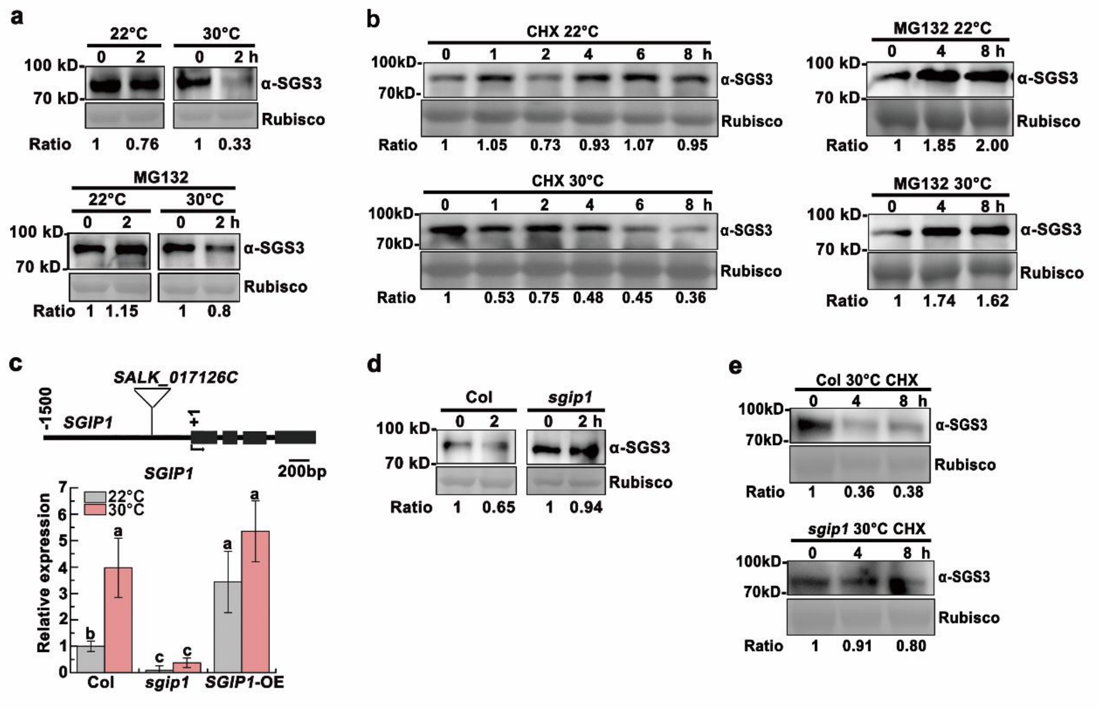
a
22°C
30°C
0
2
0
2 h
100 kD
70 kD
α-SGS3
Rubisco
Ratio 1 0.76 1 0.33
MG132
22°C
30°C
0
2
0
2 h
100 kD
70 kD
α-SGS3
Rubisco
Ratio 1 1.15 1 0.8
b
CHX 22°C
0 1 2 4 6 8 h
100kD
70kD
α-SGS3
Rubisco
Ratio 1 1.05 0.73 0.93 1.07 0.95
CHX 30°C
0 1 2 4 6 8 h
100kD
70kD
α-SGS3
Rubisco
Ratio 1 0.53 0.75 0.48 0.45 0.36
MG132 22°C
0 4 8 h
100 kD
70 kD
α-SGS3
Rubisco
Ratio 1 1.85 2.00
MG132 30°C
0 4 8 h
100 kD
70 kD
α-SGS3
Rubisco
Ratio 1 1.74 1.62
c
SALK_017126C
-1500
SGIP1
+1
200bp
SGIP1
22°C
30°C
Relative expression
0 1 2 3 4 5 6 7
b
a
c
c
a
a
Col
sgip1
SGIP1-OE
d
Col
sgip1
0
2
0
2 h
100 kD
70 kD
α-SGS3
Rubisco
Ratio 1 0.65 1 0.94
e
Col 30°C CHX
0 4 8 h
100kD
70kD
α-SGS3
Rubisco
Ratio 1 0.36 0.38
sgip1 30°C CHX
0 4 8 h
100kD
70kD
α-SGS3
Rubisco
Ratio 1 0.91 0.80

Supplementary Figure 4. Heat induces the degradation of SGS3 through the 26S proteasome pathway.

a Cell-free protein degradation assay showed the heat-enhanced degradation of SGS3. Total proteins were extracted from leaf samples of 2-week-old 22 °C or 30 °C-grown Col seedlings, and samples were incubated at 22 °C or 30 °C with or without 50 μM MG132.

b The heat-enhanced *in vivo* degradation of SGS3 in 2-week-old Col seedlings treated with 100 μM CHX or 50 μM MG132 for the indicated times.

c Analysis of the relative transcript levels of *SGIP1* in 22/30 °C-grown Col, *sgip1* and *SGIP1*-OE plants. *ACTIN2* was used to normalize expression levels. Error bars indicate the s.d. (n = 3). The position of T-DNA insertion on *SGIP1* in *SALK_017126C* was indicated. Letters indicate statistical significance based on a two-way ANOVA with Tukey's HSD post hoc analysis ($p < 0.05$).

d Cell-free degradation assay showed the delayed degradation of SGS3 in 2-week-old *sgip1* mutant grown at 22 °C compared to Col.

e *In vivo* degradation of SGS3 in 2-week-old Col and *sgip1* seedlings that were treated with 100 μM CHX for the indicated times at 30 °C. Rubisco served as a loading control and the signals were quantified (**a**, **b**, **d**, **e**). Experiments were repeated three times with similar results.
