# Supplementary material for: An H3K27me3 demethylase-HSFA2 regulatory loop orchestrates transgenerational thermomemory in Arabidopsis
Source: Cell Res. 2019 Feb 18;29(5):379–90. doi: 10.1038/s41422-019-0145-8 (PMC6796840; doi:10.1038/s41422-019-0145-8)
Supplement: Supplementary file 5 — Supplementary information, Figure S5 [file 41422_2019_145_MOESM5_ESM.pdf]

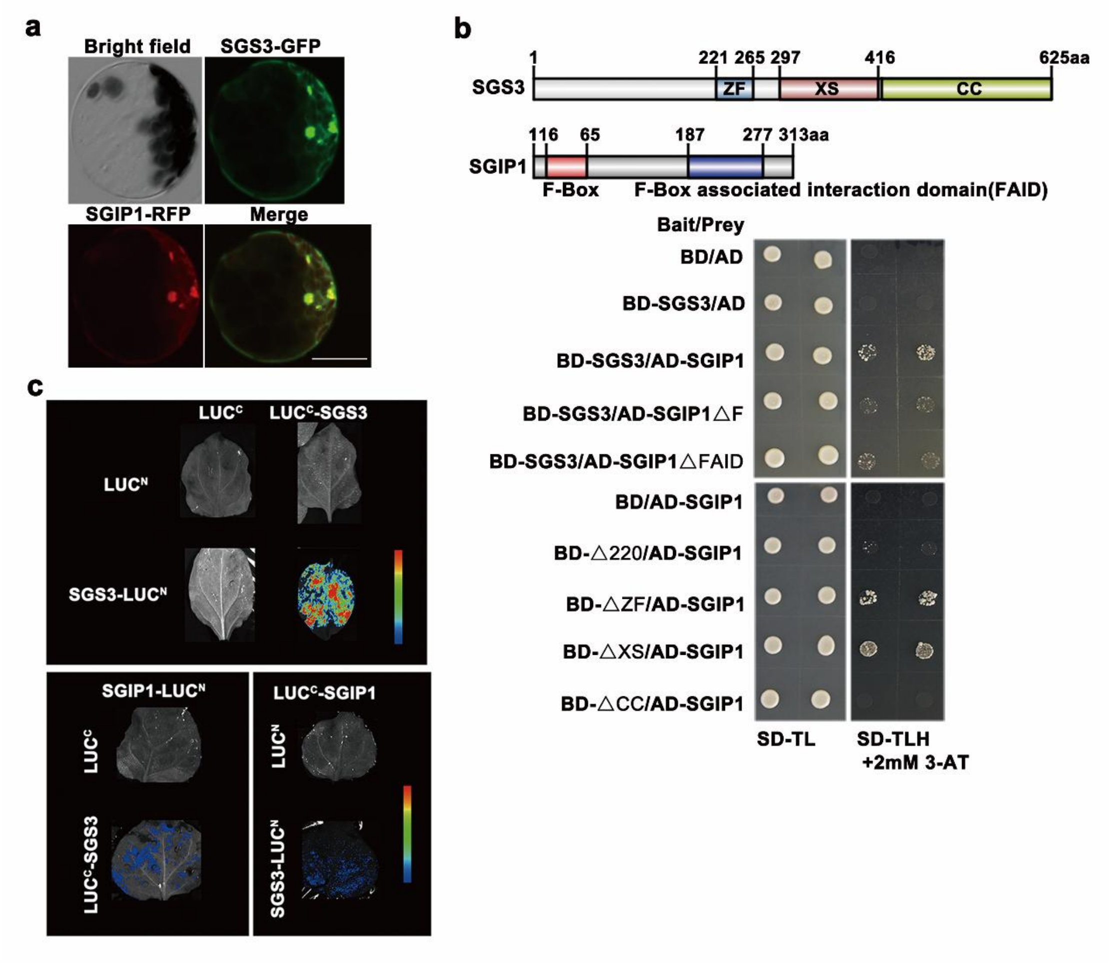
a
Bright field
SGS3-GFP
SGIP1-RFP
Merge
b
1
221
265
297
416
625aa
SGS3
ZF
XS
CC
116
65
187
277
313aa
SGIP1
F-Box
F-Box associated interaction domain(FAID)
Bait/Prey
BD/AD
BD-SGS3/AD
BD-SGS3/AD-SGIP1
BD-SGS3/AD-SGIP1△F
BD-SGS3/AD-SGIP1△FAID
BD/AD-SGIP1
BD-△220/AD-SGIP1
BD-△ZF/AD-SGIP1
BD-△XS/AD-SGIP1
BD-△CC/AD-SGIP1
SD-TL
SD-TLH
+2mM 3-AT
c
LUCᶜ
LUCᶜ-SGS3
LUCᴺ
SGS3-LUCᴺ
SGIP1-LUCᴺ
LUCᶜ-SGIP1
LUCᶜ
LUCᶜ-SGS3
LUCᴺ
SGS3-LUCᴺ

Supplementary Figure 5. SGIP1 interacts with SGS3.

a SGS3 and SGIP1 were co-localized in the cytoplasm. *Arabidopsis* protoplasts were co-transformed with SGS3-GFP and SGIP1-RFP (Scale bar, 20 μm).

b SGIP1 interacts with SGS3 in yeast two-hybrid assays. The N-terminus and CC domain of SGS3 and the F-box of SGIP1 are required. Schematics of SGS3 and SGIP1 proteins are shown.

c A split luciferase complementation assay showed the interaction of SGIP1 with SGS3 in *N. benthamiana*. In the presence of MG132, LUC signals were detected upon co-expression of LUC^{C}-SGS3+LUC^{N}-SGIP1, and of LUC^{C}-SGIP1+LUC^{N}-SGS3. Co-expression of LUC^{C}-SGS3+SGS3-LUC^{N} was used as a positive control as SGS3 acting as a homodimer[14].
