# Supplementary material for: An H3K27me3 demethylase-HSFA2 regulatory loop orchestrates transgenerational thermomemory in Arabidopsis
Source: Cell Res. 2019 Feb 18;29(5):379–90. doi: 10.1038/s41422-019-0145-8 (PMC6796840; doi:10.1038/s41422-019-0145-8)
Supplement: Supplementary file 6 — Supplementary information, Figure S6 [file 41422_2019_145_MOESM6_ESM.pdf]

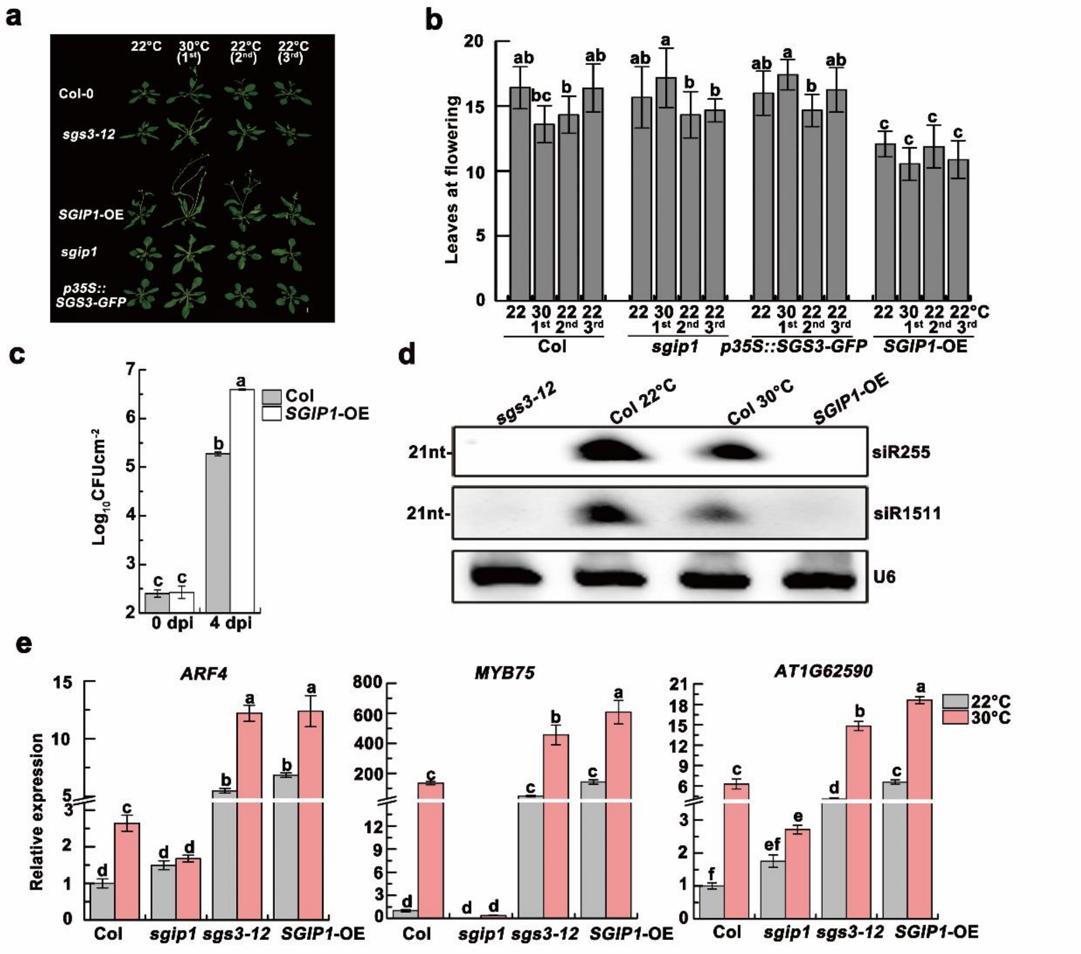
a
22°C
30°C (1st)
22°C (2nd)
22°C (3rd)
Col-0
sgs3-12
SGIP1-OE
sgip1
p35S::SGS3-GFP
b
Leaves at flowering
Col
sgip1
p35S::SGS3-GFP
SGIP1-OE
c
Log10 CFUcm-2
Col
SGIP1-OE
0 dpi
4 dpi
d
sgs3-12
Col 22°C
Col 30°C
SGIP1-OE
21nt-
siR255
siR1511
U6
e
ARF4
MYB75
AT1G62590
Relative expression
22°C
30°C
Col
sgip1
sgs3-12
SGIP1-OE

Supplementary Figure 6. Heat upregulates *SGIP1* that positively regulates tasiRNA targets.

a 24-day-old Col, *sgs3-12*, *SGIP1*-OE, *p35S::SGS3-GFP* and *sgip1* plants of the indicated generations grown at 22 °C or 30 °C. Scale bar, 1 cm.

b Flowering times of the different indicated lines as determined by leaf number (n ⩾ 15 for each line).

c *SGIP1*-OE plants were more susceptible to *Pst* DC3000(*avrRpt2*) relative to wild-type plants.

d RNA blot analysis of siR255 and siR1511 in 22/30 °C-grown Col, 22 °C-grown *sgs3-12* and *SGIP1*-OE plants. U6 was used as a loading control.

e Transcript levels of the tasiRNA targets *ARF4* (*AT5G60450*), *MYB75* (*AT1G56650*) and *AT1G62590* in 22/30 °C-grown Col, *sgip1*, *sgs3-12* and *SGIP1-OE* plants, detected by qRT-PCR. *ACTIN2* was used to normalize expression levels. Error bars indicate the s.d. (n = 3).

Letters indicate statistical significance based on a two-way (**b**, **c**, **e**) ANOVA with Tukey's HSD post hoc analysis ($p < 0.05$).
