# Supplementary material for: An H3K27me3 demethylase-HSFA2 regulatory loop orchestrates transgenerational thermomemory in Arabidopsis
Source: Cell Res. 2019 Feb 18;29(5):379–90. doi: 10.1038/s41422-019-0145-8 (PMC6796840; doi:10.1038/s41422-019-0145-8)
Supplement: Supplementary file 7 — Supplementary information, Figure S7 [file 41422_2019_145_MOESM7_ESM.pdf]

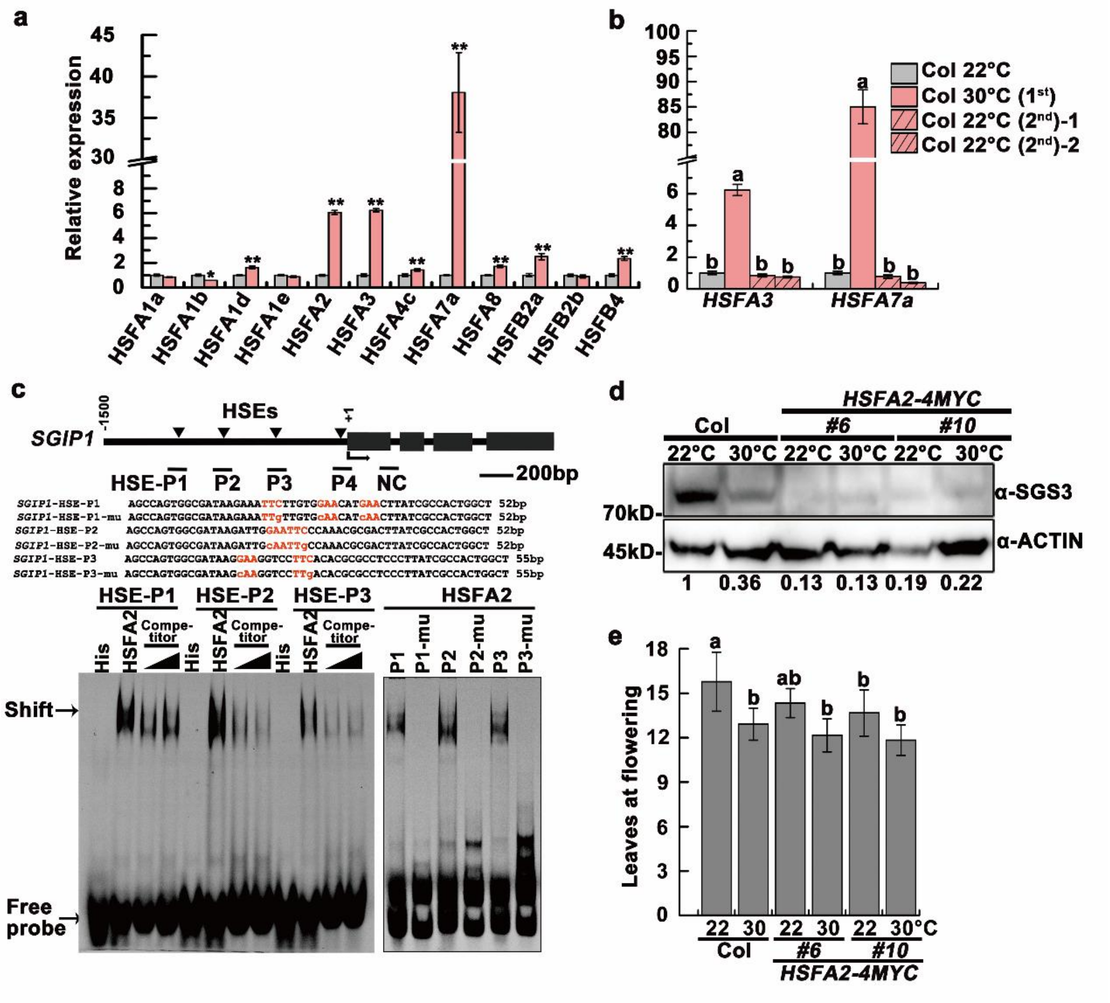

a
Relative expression
45
40
35
30
8
6
4
2
0
HSFA1a
HSFA1b
HSFA1d
HSFA1e
HSFA2
HSFA3
HSFA4c
HSFA7a
HSFA8
HSFB2a
HSFB2b
HSFB4
b
Col 22°C
Col 30°C (1st)
Col 22°C (2nd)-1
Col 22°C (2nd)-2
HSFA3
HSFA7a
c
SGIP1
-1500
HSEs
+1
HSE-P1
P2
P3
P4
NC
200bp
SGIP1-HSE-P1 AGCCAGTGGCGATAAGAAATTCTTGTGGAACATGAACTTATCGCCACTGGCT 52bp
SGIP1-HSE-P1-mu AGCCAGTGGCGATAAGAAATTgTTGTGcAACATcAACTTATCGCCACTGGCT 52bp
SGIP1-HSE-P2 AGCCAGTGGCGATAAGATTGGAATTCCCAAACGCGACTTATCGCCACTGGCT 52bp
SGIP1-HSE-P2-mu AGCCAGTGGCGATAAGATTGcAATTgCCAAACGCGACTTATCGCCACTGGCT 52bp
SGIP1-HSE-P3 AGCCAGTGGCGATAAGGAAGGTCCTTCACACGCGCCTCCCTTATCGCCACTGGCT 55bp
SGIP1-HSE-P3-mu AGCCAGTGGCGATAAGcAAGGTCCTTgACACGCGCCTCCCTTATCGCCACTGGCT 55bp
HSE-P1
HSE-P2
HSE-P3
HSFA2
His
HSFA2
Compe-titor
P1
P1-mu
P2
P2-mu
P3
P3-mu
Shift
Free probe
d
HSFA2-4MYC
Col
#6
#10
22°C 30°C 22°C 30°C 22°C 30°C
70kD-
45kD-
α-SGS3
α-ACTIN
1 0.36 0.13 0.13 0.19 0.22
e
Leaves at flowering
18
15
12
9
6
3
0
22 30 22 30 22 30°C
Col
#6
#10
HSFA2-4MYC

Supplementary Figure 7. Heat induces transgenerational accumulation of HSFA2 and overexpression of HSFA2 activates *SGIP1* expression and promotes SGS3 degradation.

a Transcript levels of *HSFA1a*, *HSFA1b*, *HSFA1d*, *HSFA1e*, *HSFA2*, *HSFA3*, *HSFA4c*, *HSFA7a*, *HSFA8*, *HSFB2a*, *HSFB2b* and *HSFB4* in 22 °C and 30 °C-grown Col, as detected by qRT-PCR. Asterisks indicate significant difference (Student's *t* test; $*p < 0.05$, $**p < 0.01$).

b Transcript levels of *HSFA3* and *HSFA7a* in 24-day-old 22 °C and 30 °C-grown Col, and two 2nd unstressed progeny lines, indicating no transgenerational upregulation of the genes. *ACTIN2* was analyzed as an internal control (**a**, **b**).

c EMSA assay shows the direct binding of HSFA2 to the HSE1-3 regions of *SGIP1*. Sequence of the probes were shown and the motif is highlighted in red. The arrow indicates the shifted bands. Excess unlabeled probe (500 ng and 1000 ng) outcompeted the labeled probe. The mutant HSEs and a downstream fragment *SGIP1-NC* (negative control) were used as negative controls.

d Immunoblot analysis of SGS3 abundance in 22 or 30 °C-grown Col, *HSFA2-4MYC#6* and *#10*. ACTIN served as a loading control and the signals were quantified.

e Flowering time of 22 °C or 30 °C-grown Col and two representative transgenic *HSFA2-MYC* lines (*#6* and *#10*). Significant difference was determined by one-way (**b**) or two-way (**e**) ANOVA with Tukey's HSD post hoc test (significance set at $p < 0.05$).
