# Supplementary material for: An H3K27me3 demethylase-HSFA2 regulatory loop orchestrates transgenerational thermomemory in Arabidopsis
Source: Cell Res. 2019 Feb 18;29(5):379–90. doi: 10.1038/s41422-019-0145-8 (PMC6796840; doi:10.1038/s41422-019-0145-8)
Supplement: Supplementary file 8 — Supplementary information, Figure S8 [file 41422_2019_145_MOESM8_ESM.pdf]

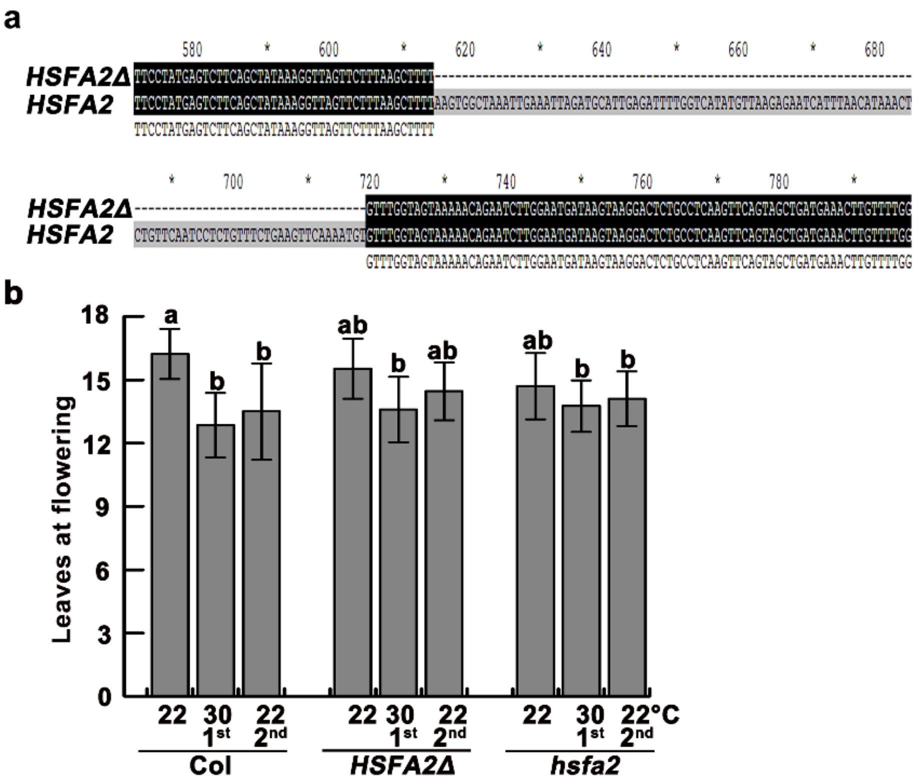
a
HSFA2Δ
HSFA2
HSFA2Δ
HSFA2
b
Leaves at flowering
18
15
12
9
6
3
0
a
b
b
ab
b
ab
ab
b
b
22
30 1st
22 2nd
22
30 1st
22 2nd
22
30 1st
22°C 2nd
Col
HSFA2Δ
hsfa2

Supplementary Figure 8. The genotype and phenotype of *HSFA2Δ* and *hsfa2* mutant plants.

a The alignment of *HSFA2* and *HSFA2Δ* genomic DNA sequences.

b Flowering times of different genotypes as determined by leaf number (n ≥ 15 for each line). Letters indicate statistical significance based on a two-way ANOVA with Tukey's HSD post hoc analysis ($p < 0.05$).
