# Supplementary material for: An H3K27me3 demethylase-HSFA2 regulatory loop orchestrates transgenerational thermomemory in Arabidopsis
Source: Cell Res. 2019 Feb 18;29(5):379–90. doi: 10.1038/s41422-019-0145-8 (PMC6796840; doi:10.1038/s41422-019-0145-8)
Supplement: Supplementary file 9 — Supplementary information, Figure S9 [file 41422_2019_145_MOESM9_ESM.pdf]

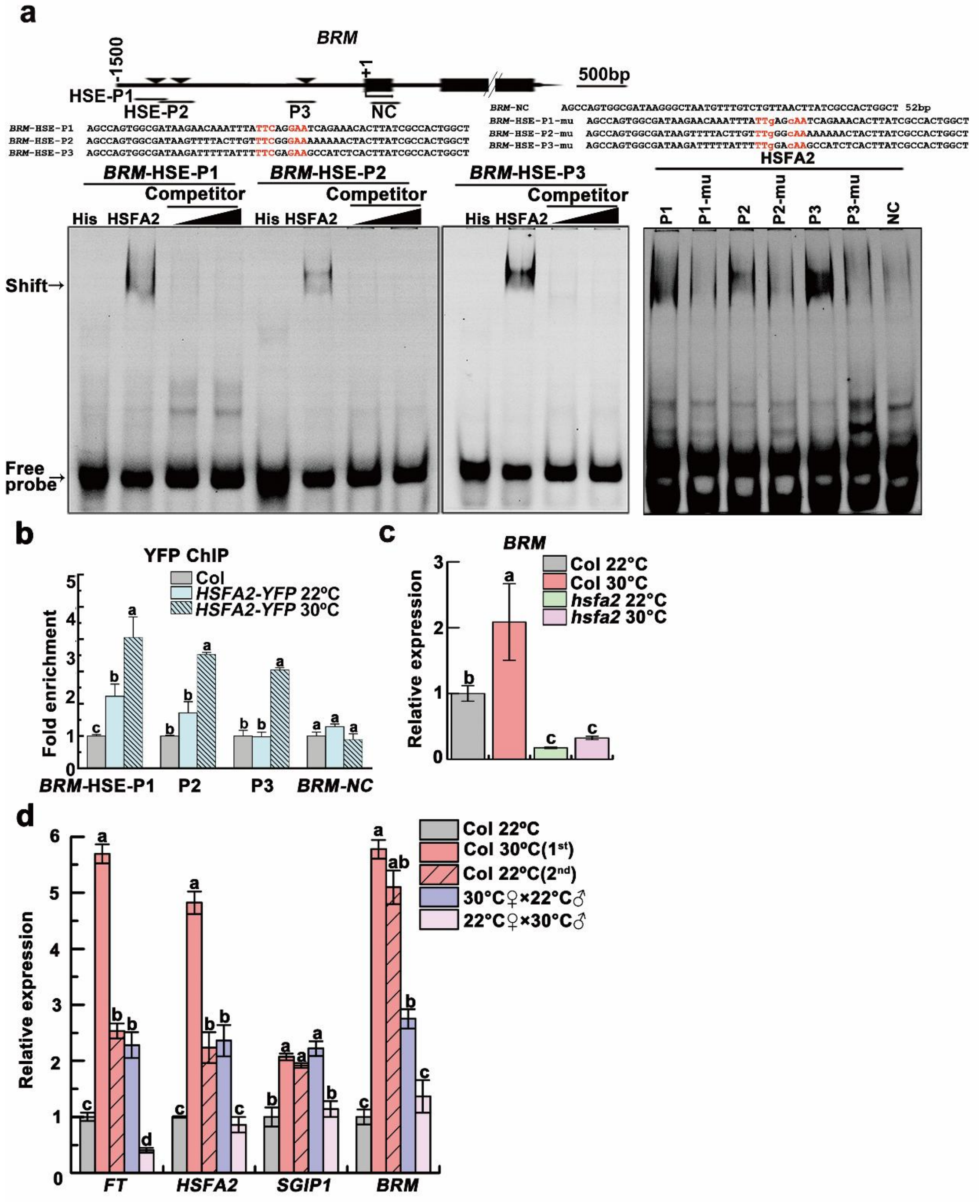
a
BRM
-1500
+1
500bp
HSE-P1
HSE-P2
P3
NC
BRM-HSE-P1 AGCCAGTGGCGATAAGAACAAATTTATTCAGGAATCAGAAACACTTATCGCCACTGGCT
BRM-HSE-P2 AGCCAGTGGCGATAAGTTTTACTTGTTTCGGGAAAAAAAACTACTTATCGCCACTGGCT
BRM-HSE-P3 AGCCAGTGGCGATAAGATTTTTATTTTTCGAGAAGCCATCTCACTTATCGCCACTGGCT
BRM-NC AGCCAGTGGCGATAAGGGCTAATGTTTGTCTGTTAACTTATCGCCACTGGCT 52bp
BRM-HSE-P1-mu AGCCAGTGGCGATAAGAACAAATTTATTgAGcAATCAGAAACACTTATCGCCACTGGCT
BRM-HSE-P2-mu AGCCAGTGGCGATAAGTTTTACTTGTTTgGGcAAAAAAAACTACTTATCGCCACTGGCT
BRM-HSE-P3-mu AGCCAGTGGCGATAAGATTTTTATTTTTgGAcAAGCCATCTCACTTATCGCCACTGGCT
BRM-HSE-P1
BRM-HSE-P2
BRM-HSE-P3
Competitor
His HSFA2
HSFA2
P1 P1-mu P2 P2-mu P3 P3-mu NC
Shift→
Free probe→
b
YFP ChIP
Col
HSFA2-YFP 22°C
HSFA2-YFP 30°C
Fold enrichment
BRM-HSE-P1 P2 P3 BRM-NC
c
BRM
Col 22°C
Col 30°C
hsfa2 22°C
hsfa2 30°C
Relative expression
d
Col 22°C
Col 30°C(1st)
Col 22°C(2nd)
30°C♀×22°C♂
22°C♀×30°C♂
Relative expression
FT HSFA2 SGIP1 BRM

Supplementary Figure 9. Heat-induced activation of *HSFA2* triggers upregulation of *BRM* and the transgenerational upregulation of *FT*, *HSFA2*, *SGIP1* and *BRM* are transmitted maternally.

a EMSA shows that His-HSFA2 protein binds to the three HSEs of *BRM* promoter *in vitro*. Excess unlabeled probes could outcompete the labeled probe. The motif is highlighted in red in the probe sequences. The mutant HSEs and a downstream fragment *BRM-NC* (negative control) were used as negative controls. The region validated by ChIP-qPCR in (**b**) is marked by a bar.

b ChIP-qPCR validation of HSFA2 occupancy at the *BRM* promoter region. The data were normalized to the corresponding input fraction. The *BRM-NC* locus was used as a negative control.

c qRT-PCR analysis of *BRM* transcript levels in 24-day-old Col and *hsfa2* grown at 22 °C and 30 °C.

d The progeny derived from Col 30 °C♀×Col 22 °C♂ but not Col 22 °C♀×Col 30 °C♂ accumulated higher transcript levels of *FT*, *HSFA2*, *SGIP1* and *BRM*. Data were shown as means ± s.d. from three replicates (**b-d**). Lowercase letters indicate statistical significance based on one-way (**b**, **d**) or two-way (**c**) ANOVA with Tukey's HSD post hoc analysis ($p < 0.05$).
