# Supplementary material for: An H3K27me3 demethylase-HSFA2 regulatory loop orchestrates transgenerational thermomemory in Arabidopsis
Source: Cell Res. 2019 Feb 18;29(5):379–90. doi: 10.1038/s41422-019-0145-8 (PMC6796840; doi:10.1038/s41422-019-0145-8)
Supplement: Supplementary file 10 — Supplementary information, Figure S10 [file 41422_2019_145_MOESM10_ESM.pdf]

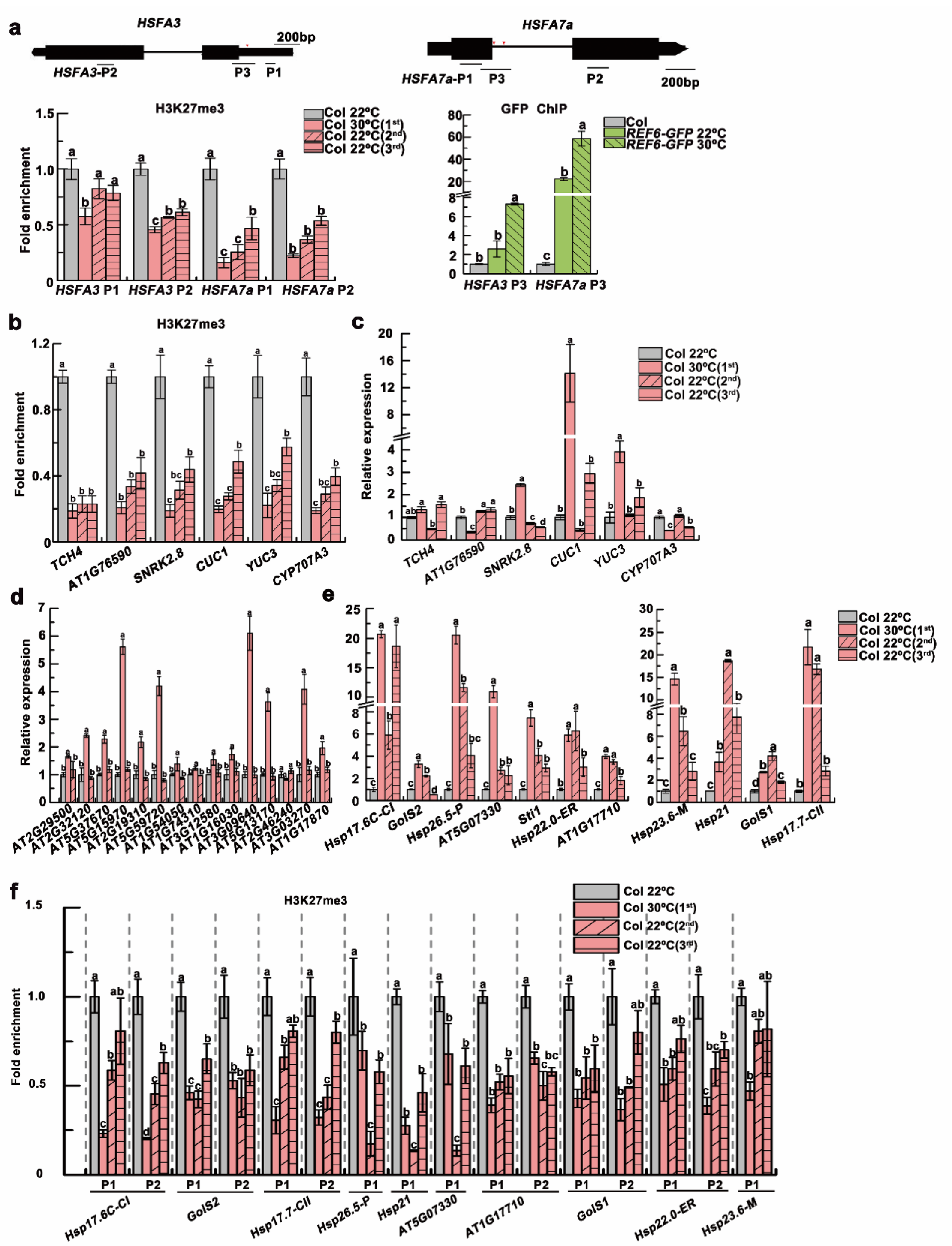
a
HSFA3
200bp
HSFA3-P2
P3
P1
HSFA7a
HSFA7a-P1
P3
P2
200bp
H3K27me3
Fold enrichment
Col 22°C
Col 30°C(1st)
Col 22°C(2nd)
Col 22°C(3rd)
HSFA3 P1
HSFA3 P2
HSFA7a P1
HSFA7a P2
GFP ChIP
Col
REF6-GFP 22°C
REF6-GFP 30°C
HSFA3 P3
HSFA7a P3
b
H3K27me3
Fold enrichment
TCH4
AT1G76590
SNRK2.8
CUC1
YUC3
CYP707A3
c
Relative expression
Col 22°C
Col 30°C(1st)
Col 22°C(2nd)
Col 22°C(3rd)
TCH4
AT1G76590
SNRK2.8
CUC1
YUC3
CYP707A3
d
Relative expression
AT2G29500
AT2G32120
AT5G37670
AT5G15970
AT2G19310
AT5G59720
AT1G54050
AT1G74310
AT3G12580
AT1G16030
AT3G09640
AT5G13170
AT2G46240
AT3G03270
AT1G17870
e
Hsp17.6C-CI
GolS2
Hsp26.5-P
AT5G07330
Sti1
Hsp22.0-ER
AT1G17710
Hsp23.6-M
Hsp21
GolS1
Hsp17.7-CII
Col 22°C
Col 30°C(1st)
Col 22°C(2nd)
Col 22°C(3rd)
f
H3K27me3
Fold enrichment
Col 22°C
Col 30°C(1st)
Col 22°C(2nd)
Col 22°C(3rd)
P1
P2
Hsp17.6C-CI
GolS2
Hsp17.7-CII
Hsp26.5-P
Hsp21
AT5G07330
AT1G17710
GolS1
Hsp22.0-ER
Hsp23.6-M

Supplementary Figure 10. The heritable REF6/BRM module-mediated H3K27me3 demethylation and *HSFA2* activation cooperate to maintain the transgenerational "ON state" of thermomemory genes.

a The H3K27me3 levels and REF6 binding detected by ChIP-qPCR at the *HSFA3* and *HSFA7a* loci. (Left) H3K27me3 levels of *HSFA3* and *HSFA7a* decreased in heat-stressed Col and unstressed progeny. (Right) ChIP-qPCR validation of REF6 binding at *HSFA3* and *HSFA7a*. Col was used as the negative control. The gene models were shown and the analyzed regions were indicated. The locations of putative REF6 binding sites were indicated by red triangles above the gene model.

b ChIP-qPCR results showed that H3K27me3 levels of other reported REF6 targets decreased in heat-stressed Col and unstressed progeny.

c qPCR analyses showed the expression of REF6 targets.

d Fifteen HSFA2 targets that were not marked by H3K27me3 enrichment were not transgenerationally upregulated by heat.

e qPCR analysis of the transcript levels of 11 HSFA2 targets, normalized to the *ACTIN2* signals.

f Analysis of H3K27me3 levels by ChIP-qPCR at 10 HSFA2 targets.

Data are shown as means ± s.d. from three replicates (**a-f**). The data was normalized to the corresponding input fraction (**a**, **b**, **f**). Lowercase letters indicate statistical significance based on one-way (**a-f**) ANOVA with Tukey's HSD post hoc analysis ($p < 0.05$). Experiments were repeated three times with similar results.
