# Supplementary material for: An H3K27me3 demethylase-HSFA2 regulatory loop orchestrates transgenerational thermomemory in Arabidopsis
Source: Cell Res. 2019 Feb 18;29(5):379–90. doi: 10.1038/s41422-019-0145-8 (PMC6796840; doi:10.1038/s41422-019-0145-8)
Supplement: Supplementary file 11 — Supplementary information, Figure S11 [file 41422_2019_145_MOESM11_ESM.pdf]

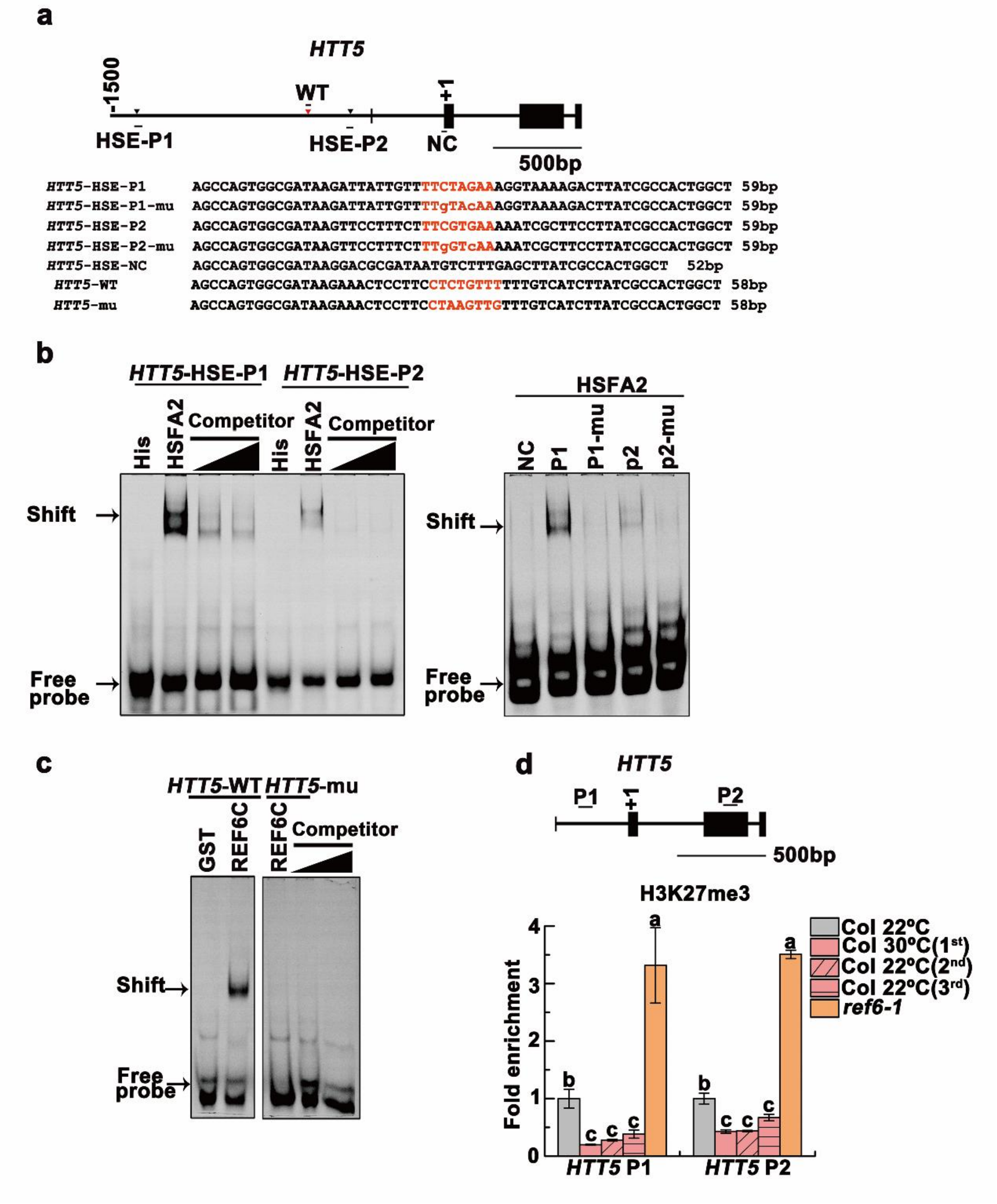
a
HTT5
-1500
WT
+1
HSE-P1
HSE-P2
NC
500bp
HTT5-HSE-P1 AGCCAGTGGCGATAAGATTATTGTTTTCTAGAAAGGTAAAAGACTTATCGCCACTGGCT 59bp
HTT5-HSE-P1-mu AGCCAGTGGCGATAAGATTATTGTTTTgTAcAAAGGTAAAAGACTTATCGCCACTGGCT 59bp
HTT5-HSE-P2 AGCCAGTGGCGATAAGTTCCTTTCTTTCGTGAAAAATCGCTTCCTTATCGCCACTGGCT 59bp
HTT5-HSE-P2-mu AGCCAGTGGCGATAAGTTCCTTTCTTTgGTcAAAAATCGCTTCCTTATCGCCACTGGCT 59bp
HTT5-HSE-NC AGCCAGTGGCGATAAGGACGCGATAATGTCTTTGAGCTTATCGCCACTGGCT 52bp
HTT5-WT AGCCAGTGGCGATAAGAAACTCCTTCCTCTGTTTTTTGTCATCTTATCGCCACTGGCT 58bp
HTT5-mu AGCCAGTGGCGATAAGAAACTCCTTCCTAAGTTGTTTGTCATCTTATCGCCACTGGCT 58bp
b
HTT5-HSE-P1
HTT5-HSE-P2
His
HSFA2
Competitor
Shift
Free probe
HSFA2
NC
P1
P1-mu
p2
p2-mu
c
HTT5-WT
HTT5-mu
GST
REF6C
Competitor
d
P1
P2
H3K27me3
Fold enrichment
Col 22ºC
Col 30ºC(1st)
Col 22ºC(2nd)
Col 22ºC(3rd)
ref6-1
HTT5 P1
HTT5 P2

**Supplementary Figure 11. *HTT5* is a co-target of REF6 and HSFA2**

**a** Schematic of the *HTT5* genomic region, black boxes indicate exons. The locations of 2 HSEs are indicated by black triangles. The CTCTGYTY motif recognized by REF6 is indicated by the red triangle. The probes used for EMSA in **b**,**c** are marked by bars. The motif is highlighted in red in the probes.

**b**,**c** EMSA shows that His-HSFA2 protein binds to the HSE motif of *HTT5* promoter (**b**) and GST-REF6C binds to the CTCTGYTY motif (**c**) of *HTT5 in vitro*. Excess wild-type unlabeled oligonucleotide could outcompete labeled probe. The mutant HSEs and a downstream fragment *HTT5-NC* (negative control) were used as negative controls.

**d** ChIP-qPCR detection of H3K27me3 levels at the *HTT5* locus. The gene model is shown at the top and the locations of the analyzed regions are indicated. Data are shown as relative fold enrichments over the background (Col). Lowercase letters indicate statistical significance based on one-way ANOVA with Tukey's HSD post hoc analysis ($p < 0.05$).
