# Supplementary material for: An H3K27me3 demethylase-HSFA2 regulatory loop orchestrates transgenerational thermomemory in Arabidopsis
Source: Cell Res. 2019 Feb 18;29(5):379–90. doi: 10.1038/s41422-019-0145-8 (PMC6796840; doi:10.1038/s41422-019-0145-8)
Supplement: Supplementary file 12 — Supplementary information, Figure S12 [file 41422_2019_145_MOESM12_ESM.pdf]

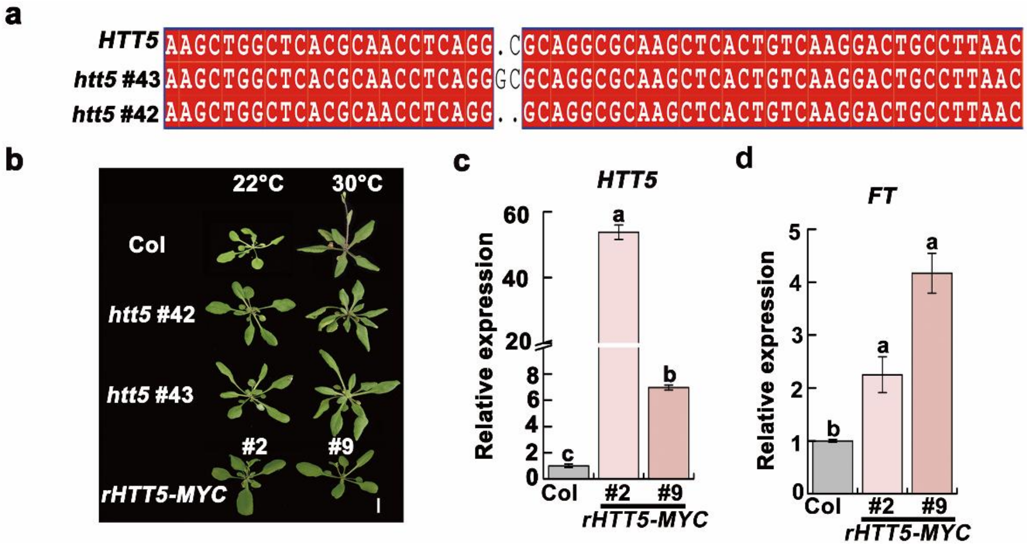
a
HTT5 AAGCTGGCTCACGCAACCTCAGG.CGCAGGCGCAAGCTCACTGTCAAGGACTGCCTTAAC
htt5 #43 AAGCTGGCTCACGCAACCTCAGGGCGCAGGCGCAAGCTCACTGTCAAGGACTGCCTTAAC
htt5 #42 AAGCTGGCTCACGCAACCTCAGG..GCAGGCGCAAGCTCACTGTCAAGGACTGCCTTAAC
b
22°C
30°C
Col
htt5 #42
htt5 #43
#2
#9
rHTT5-MYC
c
HTT5
Relative expression
Col
#2
#9
rHTT5-MYC
d
FT
Relative expression
Col
#2
#9
rHTT5-MYC

Supplementary Figure 12. *HTT5* positively regulates plant flowering but negatively regulates immunity.

a The alignment of genomic DNA sequences of the *HTT5* CRIPR/Cas9 lines (*htt5* #42 *and htt5* #43).

b 24-day-old Col and two *htt5* lines grown at 22 °C and 30 °C and two representative *rHTT5-MYC* lines grown at 22 °C. Scale bar, 1 cm.

c Analysis of the relative transcript levels of *HTT5* in 22 °C-grown Col and *rHTT5-MYC* plants.

d *FT* was upregulated in *HTT5* overexpression lines. Transcript levels were normalized to those of *ACTIN2* and data were shown as means ± s.d. from three replicates (**c, d**). Lowercase letters indicate statistical significance based on one-way (**c**, **d**) ANOVA with Tukey's HSD post hoc analysis ($p < 0.05$).
