# Supplementary material for: An H3K27me3 demethylase-HSFA2 regulatory loop orchestrates transgenerational thermomemory in Arabidopsis
Source: Cell Res. 2019 Feb 18;29(5):379–90. doi: 10.1038/s41422-019-0145-8 (PMC6796840; doi:10.1038/s41422-019-0145-8)
Supplement: Supplementary file 13 — Supplementary information, Table S1 [file 41422_2019_145_MOESM13_ESM.pdf]

Supplementary Table 1. Putative heat-responsive E3 ligases

	Subfamily	Gene ID		Subfamily	Gene ID		Subfamily	Gene ID
1	ASK	*AT2G03160*	17	F-box	*AT3G28410*	33	RING	*AT1G70910*
2	ASK	*AT3G21860*	18	F-box	*AT3G47020*	34	RING	*AT1G77830*
3	ASK	*AT3G25650*	19	F-box	*AT4G22030*	35	RING	*AT2G22690*
4	BTB	*AT2G30520*	20	F-box	*AT4G22400*	36	RING	*AT2G26000*
5	F-box	*AT1G11270*	21	F-box	*AT4G38940*	37	RING	*AT3G09770*
6	F-box	*AT1G48060*	22	PUB	*AT1G20780*	38	RING	*AT3G45510*
7	F-box	*AT1G61320*	23	PUB	*AT5G18340*	39	RING	*AT3G45540*
8	F-box	*AT1G61330*	24	RING	*AT1G10170*	40	RING	*AT4G08590*
9	F-box	*AT1G61340*	25	RING	*AT1G11100*	41	RING	*AT5G05280*
10	F-box	*AT1G77000*	26	RING	*AT1G14200*	42	RING	*AT5G06490*
11	F-box	*AT1G80440*	27	RING	*AT1G22500*	43	RING	*AT5G22920*
12	F-box	*AT2G24540*	28	RING	*AT1G26800*	44	RING	*AT5G47610*
13	F-box	*AT2G32560*	29	RING	*AT1G49220*	45	RING	*AT5G58787*
14	F-box	*AT3G13830*	30	RING	*AT1G55530*	46	PUB	*AT3G07370*
15	F-box	*AT3G18330*	31	RING	*AT1G57820*			
16	F-box	*AT3G22700*	32	RING	*AT1G60610*			
